# Xenon solubility and formation of supercritical xenon precipitates in glasses under non-equilibrium conditions

**DOI:** 10.1038/s41598-018-33556-y

**Published:** 2018-10-17

**Authors:** Anamul H. Mir, J. A. Hinks, Jean-Marc Delaye, Sylvain Peuget, S. E. Donnelly

**Affiliations:** 1 Electron Microscopy and Materials Analysis, School of Computing and Engineering, University of Huddersfield, Queensgate, Huddersfield HD1 3DH United Kingdom; 2 CEA, DEN, Laboratoire d’Étude des Matériaux et Procédés Actif, 30207 Bagnols-sur-Cèze, France

## Abstract

Estimates of noble gas solubility in glasses and minerals are important to understand the origin of these gases, particularly xenon, in the atmosphere. However, technical difficulties and ambiguities in quantifying the dissolved gases introduce large uncertainties in the solubility estimates. We present here the use of transmission electron microscopy (TEM) with *in-situ* noble gas ion implantation as a non-equilibrium approach for noble gas solubility estimates. Using a suitable Xe equation of state and Monte-Carlo simulations of TEM images, a clear distinction between Xe filled precipitates and empty voids is made. Furthermore, implantation-induced changes in the solubility are estimated using molecular dynamics simulations. These studies allow us to evaluate the xenon solubility of irradiated and pristine silica glasses and monitor *in-situ* the diffusion-mediated dynamics between the precipitates and voids — otherwise impossible to capture. On exceeding the solubility limit, supercritical xenon precipitates, stable at least up to 1155 K, are formed. The results highlight the high capacity of silicates to store xenon and, predict higher solubility of radiogenic xenon due to the accompanying radiation damage.

## Introduction

Studies on the solubility and diffusion of noble gases in glasses and minerals are crucial to help understand the noble gas fractionation and to trace the origin of the noble gases, particularly Xe, in the atmosphere. Such studies require high-pressure and high-temperature gas infusion experiments and precise quantification of the infused gas to estimate the number of solubility sites available within the structure. To a first approximation, gas solubility increases linearly with the gas pressure, following a Langmuir-adsorption isotherm^1,2^ followed by a plateau at higher pressures. However, precise quantification of the maximum solubility sites from gas infusion experiments is often marred by uncertainties arising from difficulties in clearly differentiating the following: surface adsorption and desorption; gas release from nano and micro bubbles; the presence of multiple phases; and actual physical and/or chemical solubility within the network of the host matrix. Such uncertainties can often lead to solubility values that differ significantly between the experiments (at times by more than an order of magnitude)^3^ or by up to a factor of four within a given experiment — depending on the characterisation method^1,4–7^. This is one of the main reasons for the importance of, and the need for, *in-situ* solubility experiments which is highlighted in recent work^4,5^. Furthermore, achieving a true solubility saturation requires very high pressures and temperatures which are often restricted by the limits of available instrumentation and technical difficulties therewith (e.g. crucible melting; decomposition assisted by metal uptake by Pt-crucibles resulting in the formation of unwanted compounds; limits on highest attainable pressures and temperatures due to cell design^5^; formation of crystalline phases^6^ etc.). Due to the lack of availability of high pressure instruments, most of the solubility experiments are performed in a pressure range of <1 GPa. True solubility saturation is rarely seen within this low-pressure range and it has been shown that solubility limits derived by fitting low-pressure solubility data (by assuming the validity of Henry’s law at high pressures) can be underestimated by up to an order of magnitude^2,3,8^ due to the invalidity of ideal gas laws at high pressures. A limited number of high-pressure infusion experiments and numerical simulations have shown that the solubility saturation in various glasses requires pressures of about 5 GPa – or even higher for pure silica (see section 4.4 in the supplementary information (SI) for more details)^2,3,6,9,10^.

Unlike the strong dependence of noble gas solubility on the pressure, the temperature dependence is known to be mild. Relaxing the high-temperature requirement due to its often very small effect on the solubility^1,7,11^, infusion experiments for solubility estimates could in principle be performed at room temperature if it were not for the very low diffusion coefficients of the heavy noble gases. However, high-pressures would still be necessary to completely fill all the available solubility sites and achieve the true solubility saturation. Therefore, from a fundamental perspective, noble gas implantation into a glass matrix to the point of saturation can be used to directly obtain the solubility estimates in native glass structures. Ion irradiation represents a non-equilibrium approach in which, rather than using high-temperatures to enhance the diffusion and high-pressures to establish the chemical equilibrium, energetic noble gas atoms are implanted into the network until solubility saturation is achieved. However, to derive the solubility limits from such studies, one must assess the impact and extent of the implantation damage on the solubility limit. More importantly, during implantation it is necessary to identify when local solubility saturation is reached. If these issues are addressed and compatibility between the equilibrium gas infusion experiments and these non-equilibrium implantation conditions is established or envisaged, then ion implantation represents a very convenient way of evaluating the solubility limits of noble gases in a wide variety of materials and thus circumvents many of the technical difficulties associated with the conventional methods.

The aim of the present study is to show that TEM with *in-situ* ion implantation can be used for real-time observation of the formation of Xe precipitates once the local solubility limit is exceeded. With the aid of Monte-Carlo simulations of TEM images and an appropriate equation of state (EOS) for Xe, the precipitates are shown to be supercritical Xe fluid inclusions which transform into voids and vice versa during the implantation and subsequent annealing. Using molecular dynamics (MD) simulations, the effect of irradiation damage on the Xe solubility limit is quantified and used to evaluate the solubility limit of implanted amorphous silica (a-SiO_2_), virgin a-SiO_2_ and of a complex alkali borosilicate glass.

## Results

### Xe implantation and precipitation

**Figure 1:**
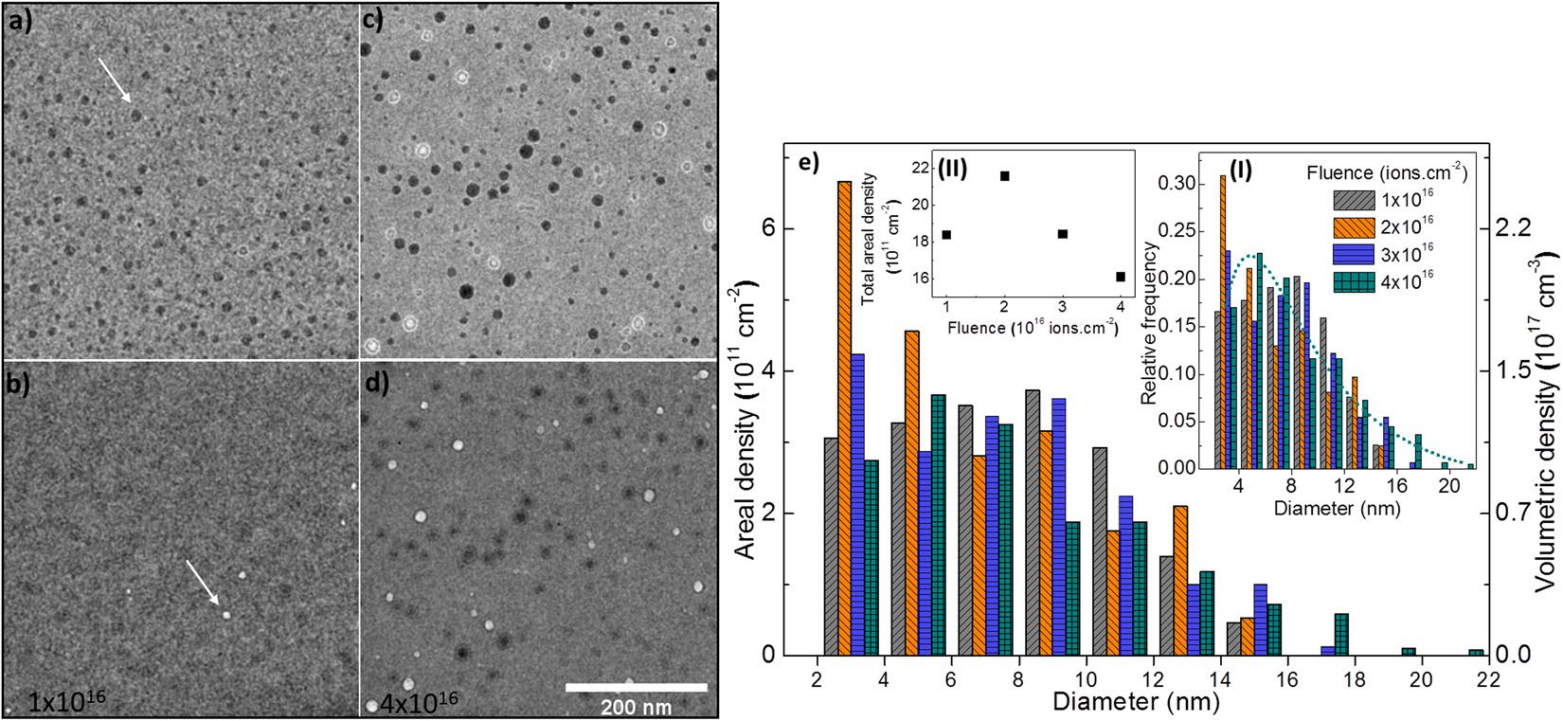
BF-TEM images and analysis of a-SiO_2_ implanted with 40 keV Xe ions at 295 K: (**a**,**b**) over and under-focus images, respectively, after implantation with 10^16^ ions.cm^−2^. The arrows indicate a precipitate and a void in (**a**,**b**) respectively (**c**,**d**) over and under-focus images, respectively, after implantation with 4 × 10^16^ ions.cm^−2^ (defocus = 2 µm for all the images (see Fig. S2.1 in the SI for the effect of image defocus on the precipitate visibility); and (**e**) areal (left-hand axis) and volumetric (right-hand axis) densities of the precipitates as functions of fluence. The insets (I) and (II) show the PD and total areal density of the precipitates as functions of fluence. Images of the pristine specimen are shown in Fig. S2.3 in the SI.

**Figure 2:**
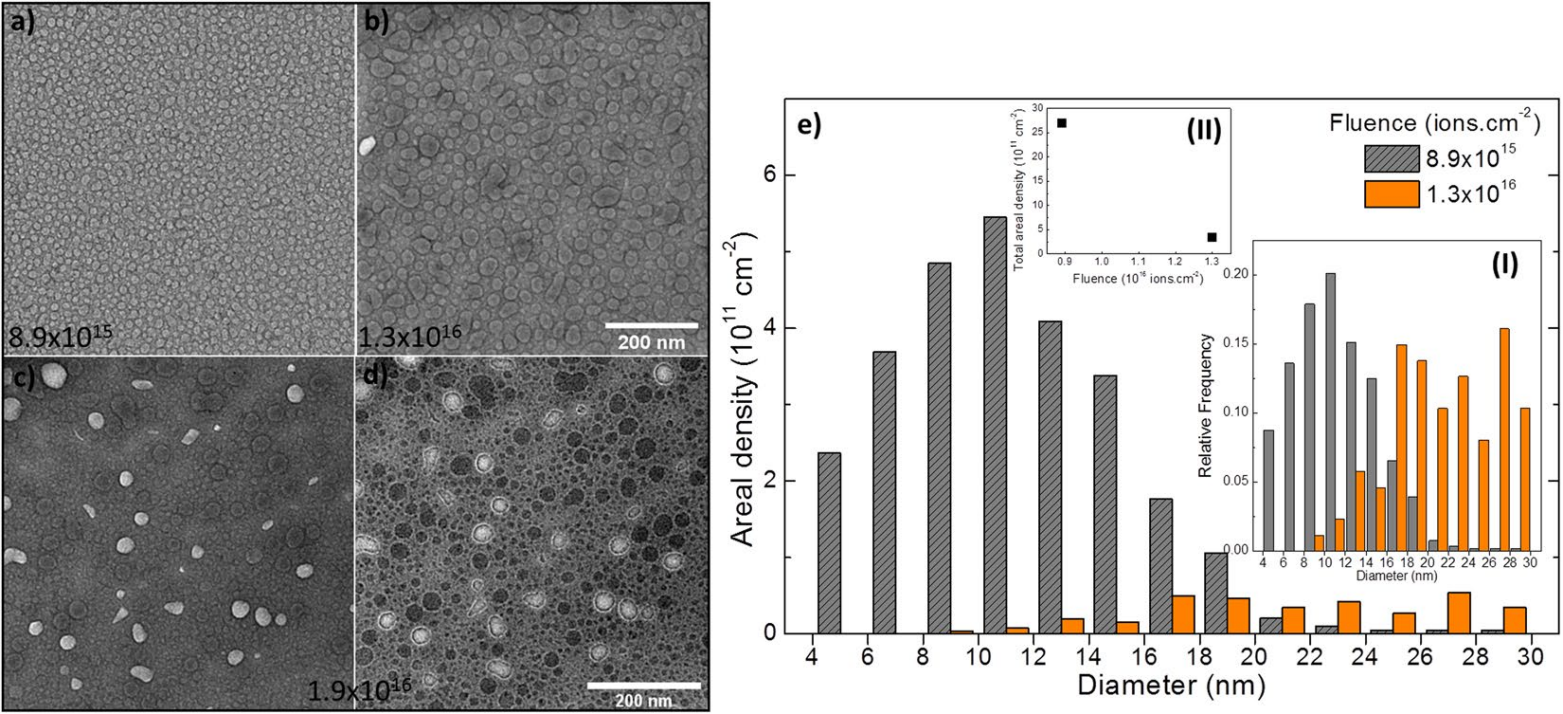
BF-TEM images and analysis of SON68 glass implanted with 45 keV Xe ions at 295 K: (**a**,**b**) under-focus images showing precipitates after implantation with 8.9 × 10^15^ and 1.3 × 10^16^ ions.cm^−2^, respectively; (**c**,**d**) under and over-focus images, respectively, showing precipitates and voids after implantation with 1.9 × 10^16^ ions.cm^−2^ (defocus = 4 µm for all the images); and (**e**) areal densities of precipitates of various diameters for the two fluences. The insets (I) and (II) show the PD and total areal density of the precipitates as functions of the fluence.

After *in-situ* implantation of a-SiO_2_ with 10^16^ Xe ions.cm^−2^ (about 2.5 × 10^21^ Xe.cm^−3^; see Fig. S1 in the SI for the ion implantation profile), distinct dark appearing features were observed in over-focus bright field TEM (BF-TEM) images as indicated by the arrow in Fig. 1(a). A few bright features were seen in under-focus imaging condition (indicated by the arrow in Fig. 1(b)). Over and under-focus images after implantation to a fluence of 4 × 10^16^ ions.cm^−2^ are shown in Fig. 1(c,d), respectively, which in addition to the dark appearing precipitates, show many bright features surrounded by circular Fresnel fringes typically associated with the bubbles/voids^12–14^ (distinct white fringes in the over-focus condition)^14^. The areal densities of the precipitates (precipitates per unit area) as function of diameter for four implantation fluences are shown in Fig. 1(e). The volumetric density, calculated by assuming that the precipitates are confined within the implanted region, is shown on the right-hand axis. The insets (I) and (II) show the probability distribution (PD) and fluence-dependence of the total areal density of the precipitates, respectively. The PD follows a typical log-normal function as shown by the dotted curve for the case of 4 × 10^16^ ions.cm^−2^ in inset (I). In the case of the alkali borosilicate glass SON68 (see Table ST1 in the SI for the composition) implanted with 45 keV Xe ions, precipitates were seen after implantation with 4 × 10^15^ ions.cm^−2^. Under-focus images of the precipitates after 8.9 × 10^15^ and 1.3 × 10^16^ ions.cm^−2^ feature in Fig. 2(a,b), respectively, and show coalescence-induced growth and the formation of non-spherical precipitates (see Fig. S2.2 in the SI for images at various fluences between these two fluence values). Precipitate distributions at these two fluences are shown in Fig. 2(e) with insets (I) and (II) showing the PD and fluence dependence of the total areal density, respectively. In comparison to a-SiO_2_, the coalescence induced shift of the PD was more dominant in the SON68 glass with a tendency to shift towards a bi-modal distribution. Under and over-focus images in Fig. 2(c,d), respectively, after implantation with 1.9 × 10^16^ ions.cm^−2^ show many voids (bright features in addition to the dark precipitates). The total areal density of the precipitates varies from 1.6 × 10^12^ to 2.2 × 10^12^ cm^−2^ for a-SiO_2_ (inset (II) in Fig. 1(e)) with small precipitates (<5 nm) in the majority. For the SON68 glass (inset (II) in Fig. 2(e)), the areal densities range from 2.6 × 10^12^ cm^−2^ at low fluence to about 4 × 10^11^ cm^−2^ at high fluence; with relatively larger precipitates (>5 nm) in the majority. These areal densities translate into a maximum surface coverage of 2–6% for a-SiO_2_ (based on four separate experiments) and 32–37% for SON68 glass (based on three separate experiments). Under irradiation, precipitate growth leading to coalescence as well as the formation of voids and their subsequent disappearance were common features observed in both the glasses. Videos SV1 and SV2 of a-SiO_2_ and SON68, respectively, which show the transformation of precipitates into voids, void coalescence and void disappearance are included in the SI. Unlike precipitates, voids, once formed, shrank in size and eventually closed upon further ion implantation. By capturing images after very small fluence increments (about 1 × 10^14^ to 2 × 10^14^ ions.cm^−2^) to follow the evolution of voids, it can be deduced that 7 ± 2 ion impacts per square nanometre of the void were required for void closure (see Fig. S3 in the SI). The void size PD is similar to that of precipitates, but at all fluences void surface coverage is always an order of magnitude less than the precipitate surface coverage. Based on four separate implantation experiments for a-SiO_2_ and three for SON68, precipitates were observed to start forming after implantation with 1.6 × 10^16^ ions.cm^−2^ ± 28% (standard error with 95% confidence interval) and 6 × 10^15^ ions.cm^−2^ ± 30% into a-SiO_2_ and SON68 glass, respectively.

### Nature of the precipitates — Monte-Carlo simulations and annealing

We exploit the Z and density dependence of the elastic scattering of electrons (see section 4.1 in the SI), Monte-Carlo simulations and, an appropriate EOS (see methods for details) to show that the observed precipitates are made of high-density supercritical Xe.

**Figure 3:**
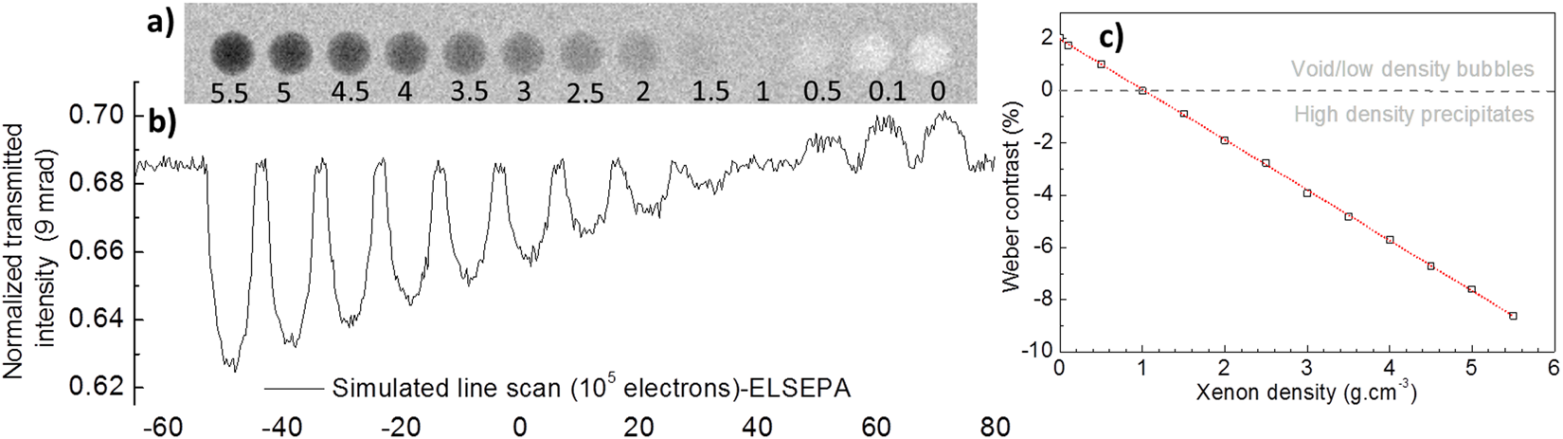
Simulation of a general at-focus TEM image showing the effect of Xe density on the contrast and transmitted electron intensity. (**a**) Simulation of 10 nm diameter Xe precipitates in a-SiO_2_ with densities ranging from 5.5 g.cm^−3^ (left) to 0 g.cm^−3^ (right); (**b**) normalized transmitted electron intensity profiles obtained by simulating a line scan using 10^5^ electrons per spot (see Methods); and (**c**) Weber contrast as a function of Xe density. The red line in (**c**) is a linear fit to the data and the horizontal black line is drawn at zero contrast observed when Xe density is about 1 g.cm^−3^.

Firstly, a general simulation highlighting the influence of Xe density on the electron transmission and image contrast is described. Figure 3(a) shows a simulated at-focus TEM image with thirteen spherical inclusions (precipitates) of 10 nm diameter in a 140 nm thick a-SiO_2_ specimen. The numbers superimposed on the simulated image are Xe densities in g.cm^−3^. The results show that the precipitates turn lighter with decreasing Xe density and eventually transform into a bright feature corresponding to a void. Supplementary Video SV3 features a simulation showing the precipitate to void transformation at a Xe release rate of 2 × 10^−18^ g.s^−1^. In general, voids and low-density precipitates (≤0.5 g.cm^−3^) appear as bright features and high-density precipitates (≥2 g.cm^−3^) appear dark. A line scan simulation showing normalized transmitted electron intensity is shown in Fig. 3(b) and Weber contrast (as defined in equation 2) calculated from this profile is shown in Fig. 3(c).

1 $$Weber\,contrast=\frac{{I}_{f}-{I}_{b}}{{I}_{b}}$$

where *I*_*f*_ and *I*_*b*_ are the transmitted intensities through precipitates/voids and a-SiO_2_, respectively. These simulations show that the contrast is proportional to the Xe density (about 1.92% per g.cm^−3^) and highlight a density range (>0.5 and <1.5 g.cm^−3^) where the Xe precipitates should become invisible. To prove this experimentally, annealing experiments aimed at slowly decreasing the precipitate density due to diffusion mediated Xe loss were performed and are presented below.

**Figure 4:**
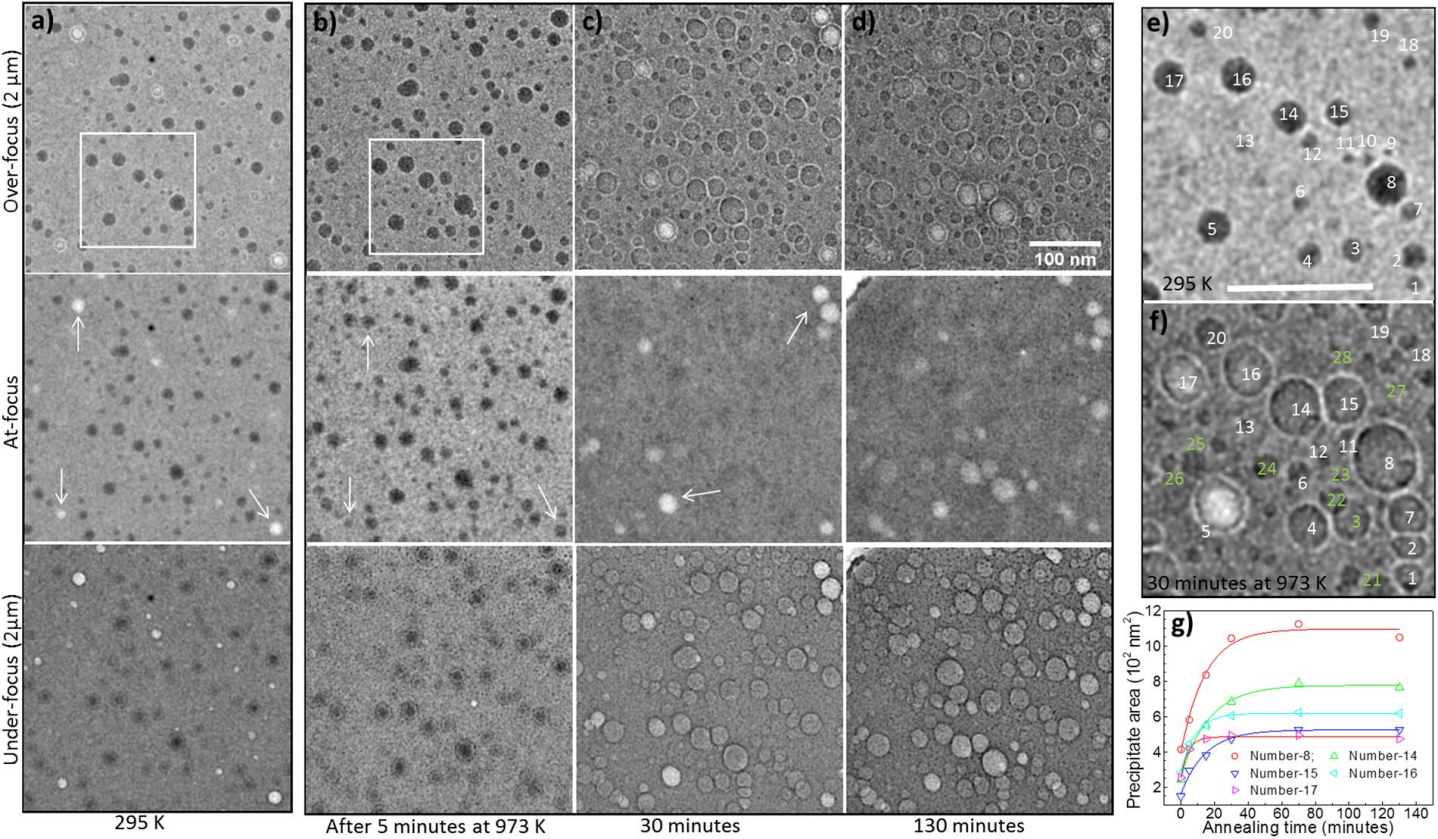
BF-TEM images showing the annealing of a-SiO_2_ implanted with 40 keV Xe ions to 4 × 10^16^ ions.cm^–2^: (**a**) TEM images at 295 K showing dark Xe-precipitates and bright voids under different focussing conditions; (**b**) after 5 minutes of annealing at 973 K; (**c**) after 30 minutes of annealing at 973 K; (**d**) after 130 minutes of annealing at 973 K; (**e**,**f**) magnified images of the region highlighted by the rectangles in (**a**,**b**), respectively; (**g**) evolution of the precipitates as a function of the annealing time at 973 K. Defocus = ±2 µm, scale marker in (**d**) applies to (**a–d**) and the scale marker in (**e**,**f**) represents 100 nm.

An a-SiO_2_ specimen implanted with 4 × 10^16^ Xe ions.cm^−2^ was annealed at 973 K. Images taken at 295 K and after 5, 30 and 130 minutes of annealing at 973 K under different focusing conditions are shown in Fig. 4(b–d), respectively. It is important to bear in mind that the Z-contrast is dominant in at-focus images, whereas, the defocussed images also feature phase-contrast. Therefore, the simulated images presented here will be compared with the at-focus BF-TEM images. Within just 5 minutes of annealing, almost all the voids — which appear as bright features at 295 K — are filled with Xe atoms and transform into dark appearing precipitates (indicated by the arrows in Fig. 4(a,b)). This indicates Xe diffusion taking place within the glass matrix and filling the empty voids during this very early stage of the annealing. From under and over-focus images, it is clear that most of the precipitates become bigger and lighter in contrast with longer annealing times (Fig. 4(c,d)). In addition, a few new voids are formed after 30 minutes of annealing (indicated by the arrows in Fig. 4(c)). Although all the precipitates can be distinguished from the background due to the presence of Fresnel fringes in the under and over-focussed images, at-focus images however indicate that most of the precipitates disappeared after about 30 minutes of annealing. Nevertheless, with prolonged annealing at the same temperature more voids emerged. This is in excellent agreement with the simulations which predicted a density range in which the precipitates should disappear as shown in Fig. 4(a). The emergence of the voids with continued annealing indicates a diffusion-induced decrease in the precipitate density leading to the brighter contrast (as predicted by the simulations for densities <0.5 g.cm^−3^ in Fig. 3(a)). It is important to note that not all of the precipitates seen in the defocused images are visible in the at-focus images. This indicates that some of them still have a Xe density that effectively gives zero contrast.

Figure 4(e,f) show the precipitates highlighted by the rectangles in Fig. 4(a,b), respectively. In Fig. 4(f), the precipitates present at 295 K are numbered from 1 to 20 (in white) and the additional precipitates which emerge only after annealing are numbered from 21 to 27 (in green). These additional precipitates were either invisible (e.g. numbers 25 and 26 as well as numerous smaller examples too small to label practically in the figure) or faintly visible (e.g. number 22) at 295 K. The size evolution of some of the precipitates as a function of the annealing time is shown in Fig. 4(g). Equilibrium sizes were attained after about 30 minutes of annealing at 973 K. The sizes of these precipitates and the densities calculated using the Xe EOS at 295 K for Fig. 4(e) and at 973 K for Fig. 4(f) are given in Table ST2 in the SI. Monte-Carlo TEM image simulations for these two cases are presented below.

**Figure 5:**
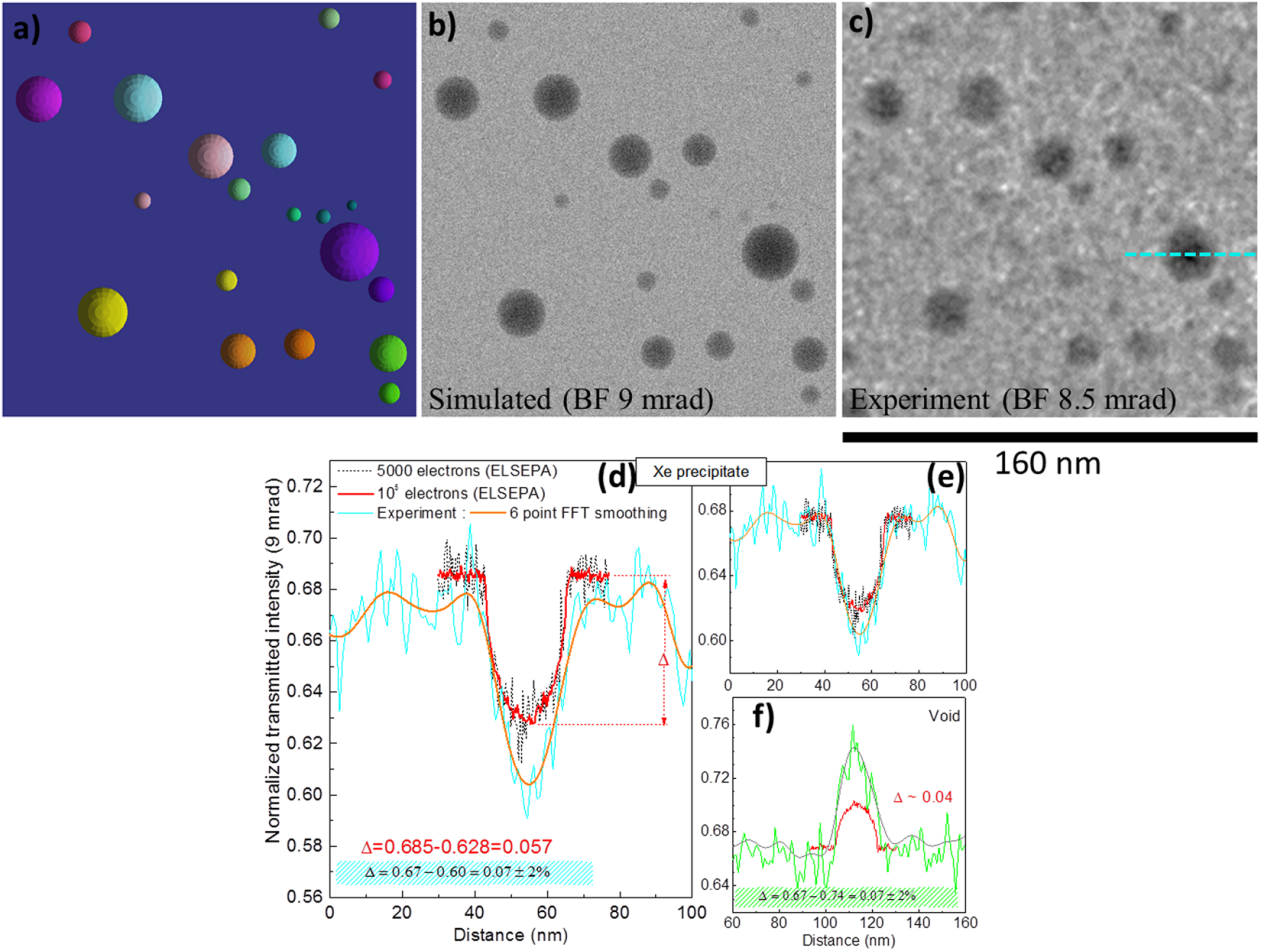
Monte Carlo TEM image simulation of Xe precipitates in a-SiO_2_ at 295 K and comparison with experiment: (**a**) top view of the simulation box; (**b**) BF-TEM image simulated using 5000 electrons per spot; (**c**) at-focus experimental BF-TEM image; (**d**) experimental transmitted electron intensity profile along the line (cyan) shown in (**c**), 6-point FFT data smoothing filter (orange) and simulated line scans for 5000 (dotted black line) or 10^5^ (solid red line) electrons per spot, respectively; (**e**) vertically shifted simulated intensity profiles for easier visual comparison; and (**f**) experimental (green line), 6-point FFT data smoothing filter (grey line) and simulated profiles (red line) across the 20 nm diameter void seen at the lower right corner in Fig. 4(a).

A visualisation of the region modelled in the Monte Carlo TEM image simulations containing Xe precipitates embedded inside a-SiO_2_ matrix is shown in Fig. 5(a). The precipitates present near the lower left corner in Fig. 4(e) were excluded from the simulations. The corresponding simulated image and the experimental at-focus image are shown in Fig. 5(b,c), respectively. All the precipitates seen in the experimental image were reproduced in the simulated image. This indicates a qualitative validation of the densities of various precipitates evaluated from the Xe EOS. For a quantitative comparison of the simulated and experimental images, a vacuum-normalized transmitted intensity profile (see Methods for details) taken along the dotted line (in cyan with a 6-point FFT data smoothing filter in orange) shown in Fig. 5(c) is compared with the simulated line scans. The line scans were simulated using 5000 electrons per spot (dotted black line) and 10^5^ electrons per spot (solid red line). The difference, ∆, between the intensity transmitted through a-SiO_2_ (background) and the centre of the Xe-precipitates (indicated by the red dotted lines in Fig. 5(d)) is also indicated in Fig. 5(d). The simulations predict an intensity drop of about 5.7% which compares reasonably well with the experimental value of 7 ± 2%. For a better relative visualization, simulated intensity profiles shifted vertically to match the mean transmission through a-SiO_2_ are shown in Fig. 5(e). Figure 5(f) shows experimental and vertically translated simulated intensity profiles of the 20 nm diameter void present near the lower-right corner indicated by the arrow in Fig. 4(a). In this case, simulated profiles seem to underestimate the electron transmission through the void. Auxiliary simulations (not shown) indicated that the observed peak transmission would better correspond to a 30 nm void. Since the void diameter is only 20 nm, this indicates that a prolate spheroid with a minor axis of 20 nm along the specimen plane and a major axis of about 30 nm perpendicular to the specimen plane would better reproduce the simulated results. Thus, the voids may actually be extended in the plane perpendicular to the specimen. Attempts to tilt the specimen to better probe the geometry of the voids failed due to their high sensitivity to the electron beam; before good imaging conditions could be achieved, the voids had significantly deformed and most of them closed due to electron beam exposure.

**Figure 6:**
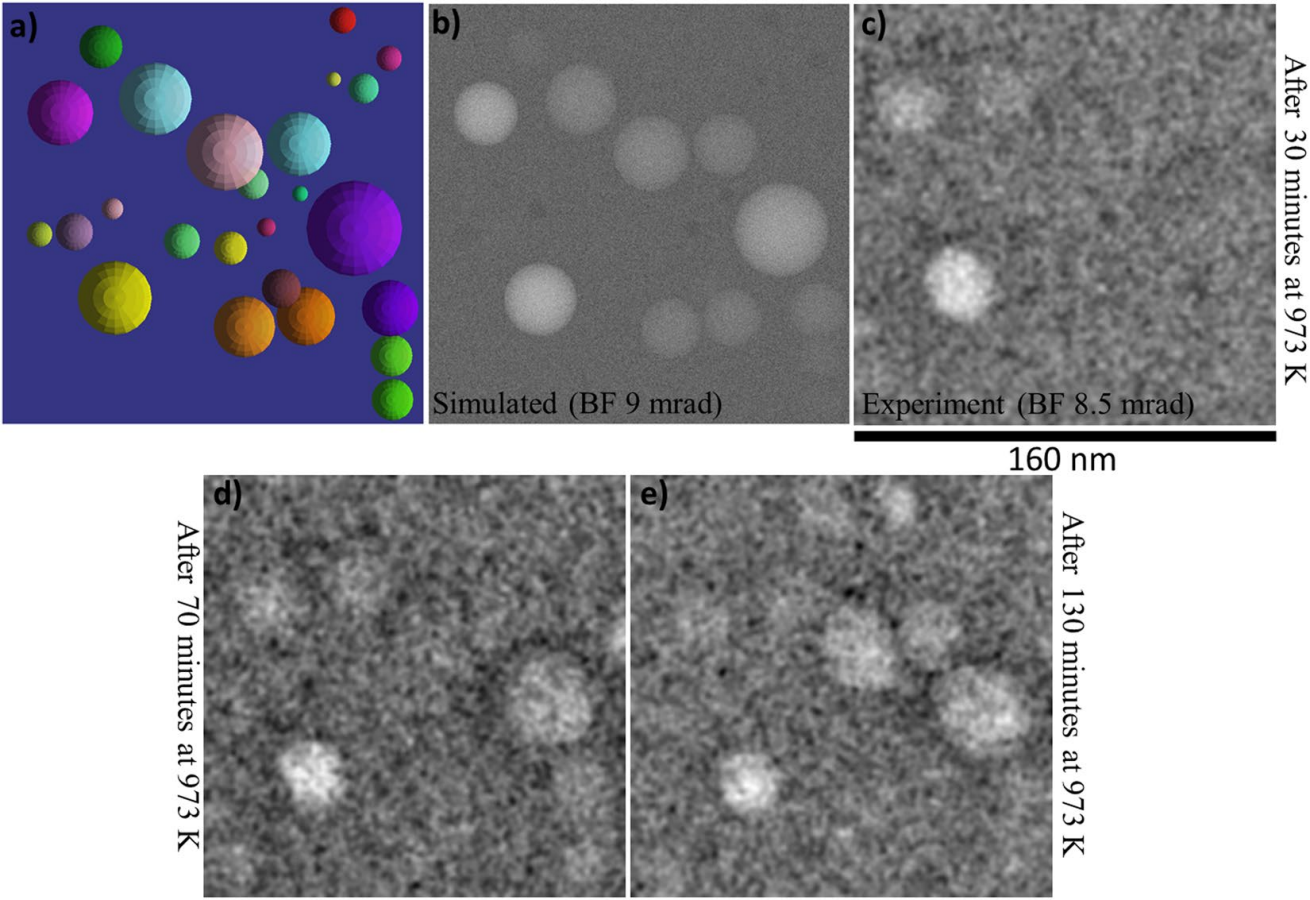
Results of Monte-Carlo TEM image simulation of Xe precipitates in a-SiO_2_ at 973 K and comparison with the experiments: (**a**) top view of the simulation box containing Xe precipitates; (**b**) BF-TEM image simulated using 5000 electrons per spot; (**c**) at-focus experimental BF-TEM image after 30 minutes of annealing at 973 K; and (**d**,**e**) at-focus experimental BF-TEM images after 70 and 130 minutes of annealing at 973 K. The scale marker below (**c**) applies to all of the images.

A comparison of the results of the image simulations with the experimental at-focus images at 973 K is presented in Fig. 6. A visualisation of the region modelled and a simulated image are shown in Fig. 6(a,b), respectively. An experimental image taken after 30 minutes of annealing is shown in Fig. 6(c) (Fig. 4(f) is the corresponding over-focus image). In addition, at-focus images after 70 and 130 minutes of annealing are shown in Fig. 6(d,e), respectively. Although only a few voids are seen in the experimental image after 30 minutes of annealing, the simulation predicts many more voids. This indicates that most of the precipitates are denser than might be assumed based on the equilibrium condition. It is interesting to note from Fig. 6(d,e) that more bright features appear as the annealing time increases. This indicates a diffusion-mediated loss of Xe from the precipitates. Also, some voids seen in Fig. 6(c) are either invisible or very faintly visible in Fig. 6(d,e)indicating a temporary densification of such precipitates. This dynamic behaviour is a result of the Xe diffusion and is consistent with Fig. 4(b) which showed a void to precipitate transformation within just five minutes of the annealing at 973 K. Therefore, even if the total number of the voids is increasing over time (indicating an overall loss of the Xe from the specimen) there is a dynamic link between the precipitates and the bulk host matrix which leads to density fluctuations during the course of the annealing. These density fluctuations currently present a problem in accurately simulating the precipitates at high-temperatures based entirely on equilibrium conditions. More predictive modelling incorporating Monte-Carlo simulation of the diffusion process could be helpful in this direction in the near future.

### Molecular dynamics simulations and the impact of ion implantation on gas solubility

**Figure 7:**
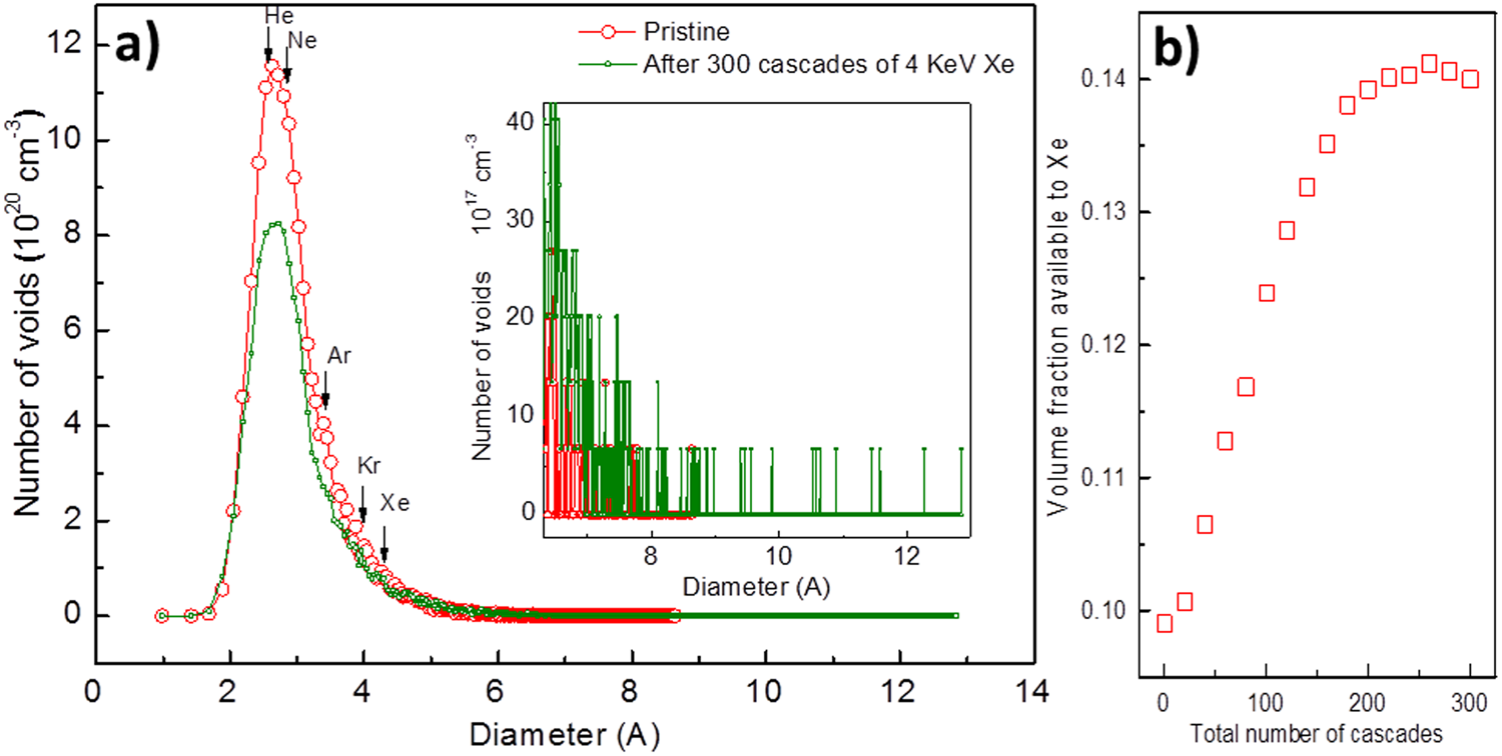
MD simulation of 4 keV Xe cascades in a-SiO_2_. (**a**) The diameters of various noble gas atoms are indicated by the arrows and the inset shows an enlarged view of the tail of the distribution; and (**b**) the variation of volume accessible to Xe as a function of the number of cascades (cumulative volume of voids with diameters larger than the Van der Waals diameter of Xe).

The effect of ion implantation on the Xe solubility was studied by simulating 300 cascades of 4 keV Xe in a-SiO_2_. The lower energy was used during the simulations to reduce the simulation time. The details of the MD simulations are given in section 4.2 in the SI. The distribution of Voronoi polyhedra as a function of the diameter of a sphere of equivalent volume^15,16^ is shown in Fig. 7(a) for undamaged and damaged a-SiO_2_ after 300 cascades. The results show an increase in the number of the larger voids at the expense of the smaller ones. After an initial increase in the volume fraction available to the Xe, saturation is observed after about 200 cascades and no further evolution up to 300 cascades (Fig. 7(b)). The results show that ion implantation can increase the number of large voids and in so doing increase the volume accessible to Xe by up to 40%. Therefore, implantation is expected to increase the Xe solubility. Delaunay tessellation^17^ was also employed to evaluate the void size distributions. The results are shown in Fig. S4 in the SI and suggest an increase in the number of the solubility sites after implantation. Due to low statistics, the number of the Xe solubility sites in the pristine a-SiO_2_ could not be evaluated accurately using Delaunay tessellation. However, from Fig. S4 in the SI, an increase is evident after the introduction of implantation damage.

### Amount of the implanted Xe

To account for the total implanted Xe, the case of a-SiO_2_ implanted with Xe to a fluence of 2 × 10^16^ ions.cm^−2^ is considered. The total number of Xe atoms in the precipitates was calculated by considering the areal precipitate distribution (Fig. 1(e)) and the precipitate densities from the Xe EOS (See Fig. S5 in the SI). Using the evaluated Xe solubility of irradiated a-SiO_2_ (4 × 10^21^ atoms.cm^−3^), the total number of Xe atoms in the precipitate-free glass volume within the implanted zone was also calculated. These two factors together account for 90% of the implanted Xe (see Table ST3 in the SI for details). This indicates that there are not a significant number of sub-nanometre Xe inclusions and therefore this provides extra confidence in the solubility estimate. It is important to emphasize that similar calculations at higher fluences will lead to erroneous estimates due to continuous precipitate-to-void transformation during ion implantation which leads to a loss of Xe from the specimen. A discussion on the gas release and the structure of voids is given in section 4.3 in the SI.

### Temperature-induced precipitate growth and coalescence

It is worth exploring the effect of the pure thermal expansion of Xe precipitates on their growth without invoking the influence of coalescence during heating. One possibility is to assume that the heating occurs under isochoric conditions and leads to over-pressurization. Another possibility is to assume that the heating process is isobaric causing a volume increase. Results of detailed calculations for both these cases are given in Table ST4 in the SI and the associated description. It is shown that for a small precipitate of 3.3 nm, thermal expansion of Xe alone suffices to explain its size at 973 K. For larger precipitates (>6 nm), 60 to 80% of the size at 973 K can be attributed to pure thermal expansion. As regards the fate of the Xe after annealing, most of it may have diffused out of the sample into the vacuum. However, there is still a significant amount of Xe left in the specimen as is evident from the relatively-light contrast of the precipitates rather than very bright contrast typical of the voids. Results of additional annealing at 1055 and 1155 K shown in Fig. S6 in the SI clearly show more voids emerging. This demonstrates that a considerable number of large low-density precipitates remain stable at least up to 1155 K and is consistent with previous studies of the thermal evolution of implanted Xe into bulk SiO_2_^18^.

### Xe solubility limit of the glasses

The fact that most of the implanted Xe is accounted for by considering the amount present in Xe precipitates and in the precipitate-free glass volume, demonstrates that the Xe solubility limit is correctly estimated. Therefore, Xe solubility for irradiated and pristine a-SiO_2_ is about 6 ± 1.7 atomic % (4 × 10^21^ ions.cm^−3^) and 3.6 ± 1.4 atomic % (2.4 × 10^21^ ions.cm^−3^), respectively. The solubility of the pristine a-SiO_2_ was simply evaluated by assuming a 40% increase due to the implantation as indicated by the MD simulations. The solubility limit for the alkali borosilicate glass on the other hand is only one-third of the Xe solubility in a-SiO_2_. This is in good agreement with the general trend that the presence of glass modifier elements decreases the gas solubility^1,2,19–21^ by partially occupying the available solubility sites themselves.

A precise quantitative comparison with the solubility data available in the literature is difficult partly due to a lack of the high-pressure solubility data for Xe; except for some high-pressure data on tholeitic^2^ and Haplogranitic^22^ compositions, and partly due to the uncertainty of up to an order of magnitude in the available solubility limits. From the few reported high-pressure experiments and the numerical simulations of noble gas solubilities in silicates^2–4,6,9,23^, it is clear that saturation is attained after pressurization by about 5 GPa. By converting the solubility data available in the literature to atoms.cm^−3^ at 5 GPa and considering that Xe solubility is about one-third that of the Ar solubility, we estimate a solubility limit of about 10^21^ atoms.cm^−3^ in SiO_2_ (see section 4.4 in the SI) under equilibrium conditions. Although the solubility limit obtained from our ion implantation results (2.4 × 10^21^ ions.cm^−3^) may seem slightly overestimated, it is nonetheless compatible with typical previously evaluated solubility values; which are known to have fluctuations up to an order of magnitude, and more recent ones showing a Xe solubility of about 1.25 at % in silica-rich hydrated Haplogranitic compositions^22^. However, Xe solubility in SiO_2_ can be expected to be higher than the Xe solubility in hydrated HPG due to the presence of H_2_O and lower silica content (~70%) in the HPG. In addition, linear extrapolation of the data up to 5 GPa may yield slightly lower values than the actual solubility due to the invalidity of Henry’s law at high pressures (see section 4.4 in the SI for details of the solubility data available in the literature).

### Conclusion

This study demonstrates that a clear distinction between dense supercritical Xe precipitates and voids can be made and that the dynamics of their inter-conversion during annealing and implantation can be tracked using *in-situ* TEM. It also highlights the potential of using Monte Carlo simulations and equation of state to provide a qualitative and quantitative validation of the TEM images of the precipitates and voids. The solubility limits obtained under non-equilibrium conditions for amorphous silica and a complex borosilicate glass clearly demonstrate a decrease in the solubility due to the addition of glass network modifiers to the latter. This is already well documented in the literature based on the equilibrium infusion experiments and is correctly observed during our non-equilibrium implantation conditions. Moreover, molecular dynamics simulations showed an increase in the number of the solubility sites due to implantation damage. These results give an important insight in that the local storage of radiogenic Xe can be expected to be higher than evaluated from any infusion experiments or numerical simulations as radiation-damage-induced solubility changes are not accounted for in such experiments. Furthermore, as precipitation of supercritical Xe is observed in TEM images upon exceeding the solubility limit, the limit obtained from ion implantation represents the maximum possible. This limit should serve as a very stringent constraint for any forthcoming numerical simulations of Xe solubilities in silica and borosilicates. Nevertheless, the high Xe solubility limits of amorphous silica and complex borosilicates clearly support the idea that a large amount of Xe can remain trapped in silicates^4,24^. The strength of ion implantation experiments clearly lies in the ability to control and account for the implanted atoms using a combination of the equation of state, numerical simulations and *in-situ* TEM images. Although ion implantation itself alters the solubility limit, this can be accounted for by considering the results of the MD simulations. To advance the MD work on the impact of implantation damage on solubility, higher energy cascades need to be simulated. Furthermore, MD simulations of ion impacts on dense Xe precipitates would be a next step in understanding the atomistic details of the gas release from Xe precipitates during implantation. In any case, implantation-induced changes are smaller than the uncertainties that arise due to ambiguities associated with quantifying the released gases from high-temperature and high-pressure gas-infused specimens. It is worth highlighting that TEM with *in-situ* ion implantation offers a great potential in performing detailed isochronal annealing studies of precipitate to void transformation and vice versa and, use such data to estimate the noble gas diffusion coefficients and activation energies.

## Methods

### Specimen preparation and ion implantation

Electron-transparent TEM specimens of amorphous silica (a-SiO_2_: Suprasil 300) and SON68 glass were prepared using an FEI Quanta 200 3D focussed ion beam system with 30 keV Ga ions and currents ranging from 300 to 50 pA. Final polishing was done at ±2 degrees. The thickness of the a-SiO_2_ specimen presented in the TEM images and simulations in this work was determined to be 140 ± 15 nm (0.55 ± 0.05 inelastic mean free paths) using electron energy loss (EELS) spectroscopy. The EELS spectra were acquired using a 300 keV electron beam and a Gatan imaging filter (GIF Quantum) attached to a Hitachi H-9500 TEM (the value of the inelastic mean free path was taken from ref.^25^). All the specimens were implanted using the MIAMI-2 TEM with *in-situ* ion irradiation facility at the University of Huddersfield, United Kingdom. Specimens were implanted at 295 K with singly-charged 40 keV Xe ions (projected implantation depth is about 27 ± 7 nm; see Fig. S1 in the Supplementary Information (SI)). Four ion implantations of four similarly-prepared a-SiO_2_ specimens (thickness between 100 to 200 nm) with fluxes ranging from 2 × 10^13^ to 4 × 10^13^ ions.cm^−2^.s^−1^ were performed. Fluences as high as 6 × 10^16^ ions.cm^−2^ (1.5 × 10^22^ Xe.cm^−3^) were attained in certain cases to follow the evolution of the Xe precipitates once formed. However, for the sake of brevity and consistency, most of the results presented in this article are from the 140 nm thick specimen implanted with 3.8 × 10^13^ ions.cm^−2^.s^−1^ up to a maximum fluence of 4 × 10^16^ ions.cm^−2^. The SON68 glass, was implanted with 45 keV Xe ions at a flux of about 2.5 × 10^13^ ions.cm^−2^.s^−1^. The electron beam was switched off during both ion implantation and annealing and was used only for imaging or for capturing short video sequences only on some specimens. For TEM imaging, the electron flux was about 8 × 10^17^ electrons.cm^−2^.s^−1^ and an objective aperture with a collection angle of 8.5 mrad was used. The images are taken after every 2 × 10^15^ Xe.cm^−2^ fluence increments and the bubble formation could have taken place at any fluence between the reported value and the previous observation.

### Specimen Annealing

Some of the implanted specimens containing Xe precipitates were annealed using a Gatan double-tilt heating holder to study the effect of the temperature on the growth, coalescence and stability of the Xe precipitates. Preparatory annealing experiments were performed at different temperatures from which it was found that the temperature-induced changes in the precipitates should occur within a reasonable time (a few hours) in the temperature range of 973 K to 1023 K. Most of the annealing was then performed at 973 K with some limited annealing at higher temperatures (maximum of 1155 K). The specimen heating rate in all cases was 100 K.min^−1^.

### Monte-Carlo Simulations

The primary motivation of this study was to provide a direct and quantitative way of addressing the observation of the Xe precipitates, voids and their inter-conversion either due to ion implantation or annealing. The image simulations presented here are based on elastic scattering of electrons from atoms using the Monte Carlo simulation code CASINO^26,27^. The simulation involves defining a beam spot size (0.6 nm diameter in the current work) and spacing between the spots (0.3 nm in the current work). The simulation then raster scans an electron beam of a predefined energy (300 keV in the current work)) and intensity across the specimen and calculates the electron scatter. The images were simulated using 5000 electrons per spot. Line scan simulations were performed using either 5 × 10^3^ (black profile in Fig. 5(d)) or 10^5^ (red profile in Fig. 5(d)) electrons per spot. **EL**astic **S**cattering of **E**lectron and **P**ositrons by **A**toms (ELSEPA) based on relativistic Dirac partial-wave calculation scattering cross-sections were used (see Fig. S7 in SI for a comparison of the results with Mott scattering cross-section). Some of the pre-requisites and how they were fulfilled are listed in section 4.5 in the SI.

#### Density and thickness of the host matrix

The host matrix in the current work was a-SiO_2_ with a density of 2.2 g.cm^−3^ and a thickness of 140 ± 15 nm. The thickness was calculated by applying the log-ratio method to the collected EELS spectrum and using an inelastic mean free path from the literature^25^ (specimen thickness is given by the product of the “inelastic mean free path” and “relative thickness” calculated from the EELS spectrum: 250 nm × 0.55 = 137 nm). However, during annealing, the specimens were observed to curl and bend as evidenced by the focus variation at the specimen edges and a progressive darkening due to a drop in the transmitted intensity. It was assumed that the centres of the precipitates remained stationary and, by measuring the distance between the centres of the precipitates, an approximate estimate of the tilt angle and effective specimen thickness was obtained. The effective specimen thickness after 30 minutes of annealing was estimated to be about 160 nm. Therefore, the image simulation at 973 K considers a specimen thickness of 160 nm.

#### Transmitted intensity profiles

Raw experimental images were read using the Fiji software^28^ and normalized by the average transmitted intensity in a vacuum region of 150 × 150 pixels (referred to as vacuum normalization). The experimental transmitted profiles were obtained by taking single-pixel-wide line-scans across a precipitate or void which were then compared with the simulated line-scans. The simulated line-scans were also normalised by dividing the transmitted by the incident intensity.

#### Density and size of the Xe-precipitates and their evolution with temperature

The Xe-precipitates were assumed to be spherical. Their sizes (projected areas and diameters) were determined using the FIJI software^28^. Circles were manually fitted to the precipitates imaged in over-focus conditions as shown in Fig. 4(e,f) to measure the diameters at 295 and 973 K, respectively. The various precipitates numbered in these two images were considered in the simulations. A few precipitates near the boundaries were excluded. Prior to this, one separate simulation was performed in which the precipitates were placed at different positions within the matrix to see the effect of precipitate depth on the final image. No depth dependent effect was observed making this an arbitrary parameter. The top surface of the precipitates was then considered to be at a depth of 10 nm from the top surface of the specimen. By doing so, the precipitates were placed within the actual implanted region. To calculate the density of Xe-precipitates an appropriate equation of state is needed as described in the next section.

### Xe equation of state and the density of Xe precipitates

An equation of state (EOS) relates pressure, density and temperature (P-ρ-T) with each other. To calculate the pressure that the Xe-precipitates experience, we assume that the precipitates are in mechanical equilibrium and that the internal pressure is balanced by the surface tension^29^:

$$P=2\frac{T}{r}$$

where *P* is the internal pressure of a Xe precipitate, *T* is its surface tension of the host matrix and *r* is the precipitate radius. The values of the precipitate radii were obtained as described above by graphically fitting the precipitates with the circles. The value of the surface tension for a-SiO_2_ varies from about 0.2 to 0. 3 N.m^−1^ in the temperature range of 1270 to 2070 K^30–32^ with no evident temperature correlation. Since these values are usually obtained at high temperatures (>1270 K), a temperature correction when extrapolating to room temperature is required. a-SiO_2_ has a positive temperature coefficient (i.e. an increase in the surface tension with an increase in temperature). The temperature coefficient calculated from ref.^30^ is about 3.4 × 10^−5^ N.m^−1^.K^−1^. Therefore, the room temperature surface tension is expected to be slightly smaller than the high-temperature values. Owing to the variation in the surface tension values available in the literature, a maximum possible surface tension of 0.3 N.m^−1^ was used for the pressure calculations. It is shown in the SI that even an uncertainty of up to 30% in the surface tension values does not have a significant effect on the precipitate density. The radii of the precipitates numbered from 1–20 in Fig. 4(e) and 1–28 in Fig. 4(f) along with the corresponding pressures are shown in Table ST2 in the SI. A detailed description of Xe equations of state and Xe melting curves is given in section 4.6 in the SI. The Xe present in the precipitates at 295 K exists as inclusions of supercritical fluid (the diffraction patterns did not show any crystalline xenon. See section 4.6 in the SI for a discussion). Only precipitates with diameters greater than 210 nm will have pressures less than the Xe critical pressure and exist as a distinct gaseous phase at 295 K and above. In addition, diffusive losses at high temperatures (>800 K) will eventually lead to the formation of gaseous bubbles (at 973 K, a diffusion length of 2 nm in 10 minutes can be expected based on the Xe diffusion coefficient)^33^.

## Electronic supplementary material

SV1

SV2

SV3

Supplementary information
