# Supplementary material for: Xenon solubility and formation of supercritical xenon precipitates in glasses under non-equilibrium conditions
Source: Sci Rep. 2018 Oct 17;8:15320. doi: 10.1038/s41598-018-33556-y (PMC6192981; doi:10.1038/s41598-018-33556-y)
Supplement: Supplementary file 4 — Supplementary information [file 41598_2018_33556_MOESM4_ESM.docx]

**Supplementary information**

**Xenon solubility and formation of supercritical xenon precipitates in glasses under non-equilibrium conditions**

Anamul H Mir^a*^, J.A. Hinks^a^, Jean-Marc Delaye^b^, Sylvain Peuget^b^, S.E. Donnelly^a^

^a^ Electron Microscopy and Materials Analysis Group, School of Computing and Engineering, University of Huddersfield, Huddersfield HD1 3DH, UK

^b^ CEA, DEN, Laboratoire d’Étude des Matériaux et Procédés Actif, 30207 Bagnols-sur-Cèze, France

*^*^ Corresponding author: mirinamulhaq@gmail.com*

------------------------------------------------------------------------------------------------------------------------------------------------------

**Section-1: Supplementary images**

------------------------------------------------------------------------------------------------------------------------------------------------------

***Figure S1****: Implantation profile of 40 keV xenon ions multiplied by xenon precipitate formation threshold (~ 1.6x10^16^ ions.cm^-2^ for a-SiO_2_) (obtained from SRIM software[1]). Vertical black lines show integration limits used to calculate average implantation concentration (limits are at one-tenth of the peak value).*

------------------------------------------------------------------------------------------------------------------------------------------------------


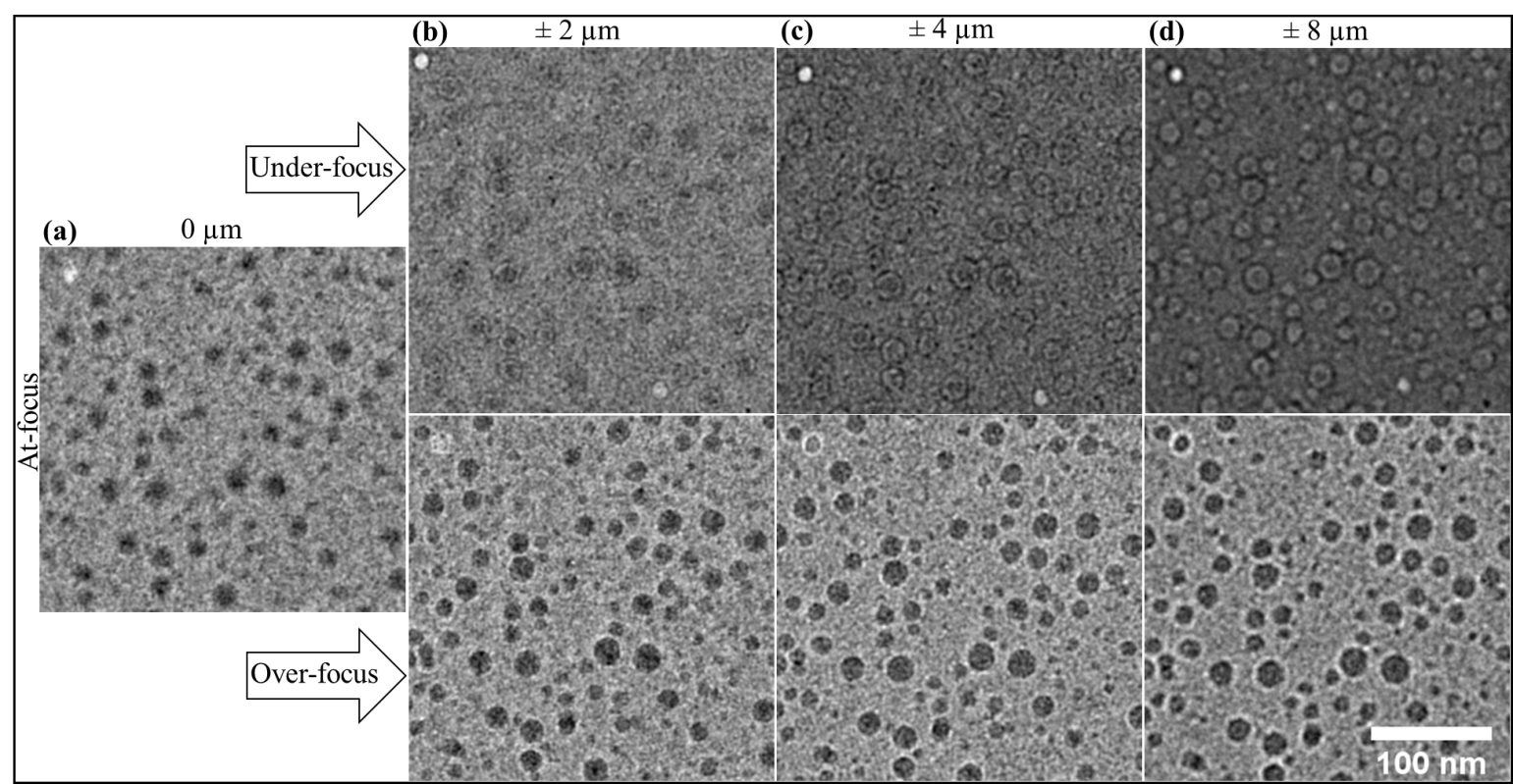


***Figure S2.1:*** *Bright field TEM images of xenon implanted a-SiO_2_ at various defocus values.****(a)****, At-focus image. The dark spots are xenon precipitates.* ***(b, c, d)****, Under (top) and over-focus (bottom) images at 2 µm, 4 µm and 8 µm image defocus respectively.*

**Additional details:** Specimen = a-SiO_2_ (different from the one shown in the main article), Thickness = 175 nm (Measured after ion implantation), Xe ion energy = 40 keV, Fluence= 6.4x10^16^ ions.cm^-2^, implantation temperature = 295 K, Electron beam energy = 300 keV, Objective aperture = 8.5 mrad, Magnification = 50000, Image exposure time = 0.5 s, electron flux ~ 8000 electrons.nm^-2^.s^-1^, images shown above were taken 6 months after the implantation.

**
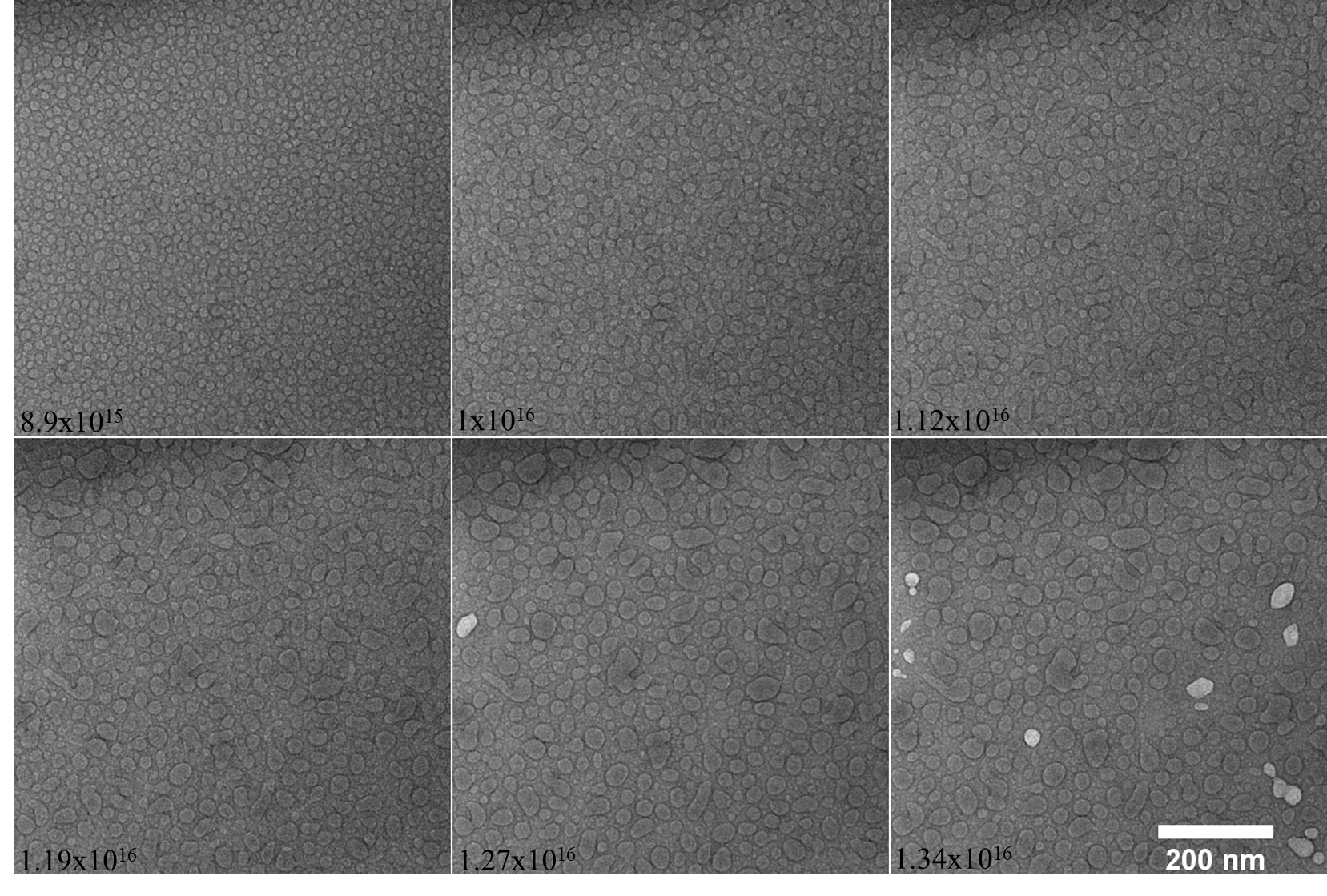
**

***Figure S2.2:*** *Bright field TEM images of xenon precipitates in SON68 glass after irradiation with 45 keV Xe ions. The fluences (in ions.cm^-2^) are shown on each image. The fluence of 1.27x10^16^ is rounded off to 1.3 x10^16^ in the main article. The white features starting to appear after 1.27x10^16^ ions.cm^-2^ are voids. (Image defocus= 4 µm under-focus).*


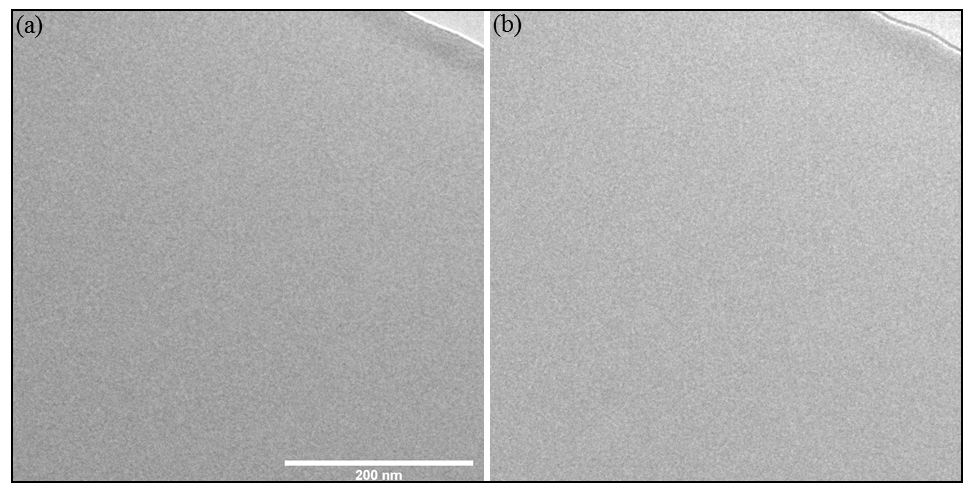


*Fig. S2.3: BF-TEM image of the a-SiO_2_ specimen before Xe ion implantation.*

------------------------------------------------------------------------------------------------------------------------------------------------------


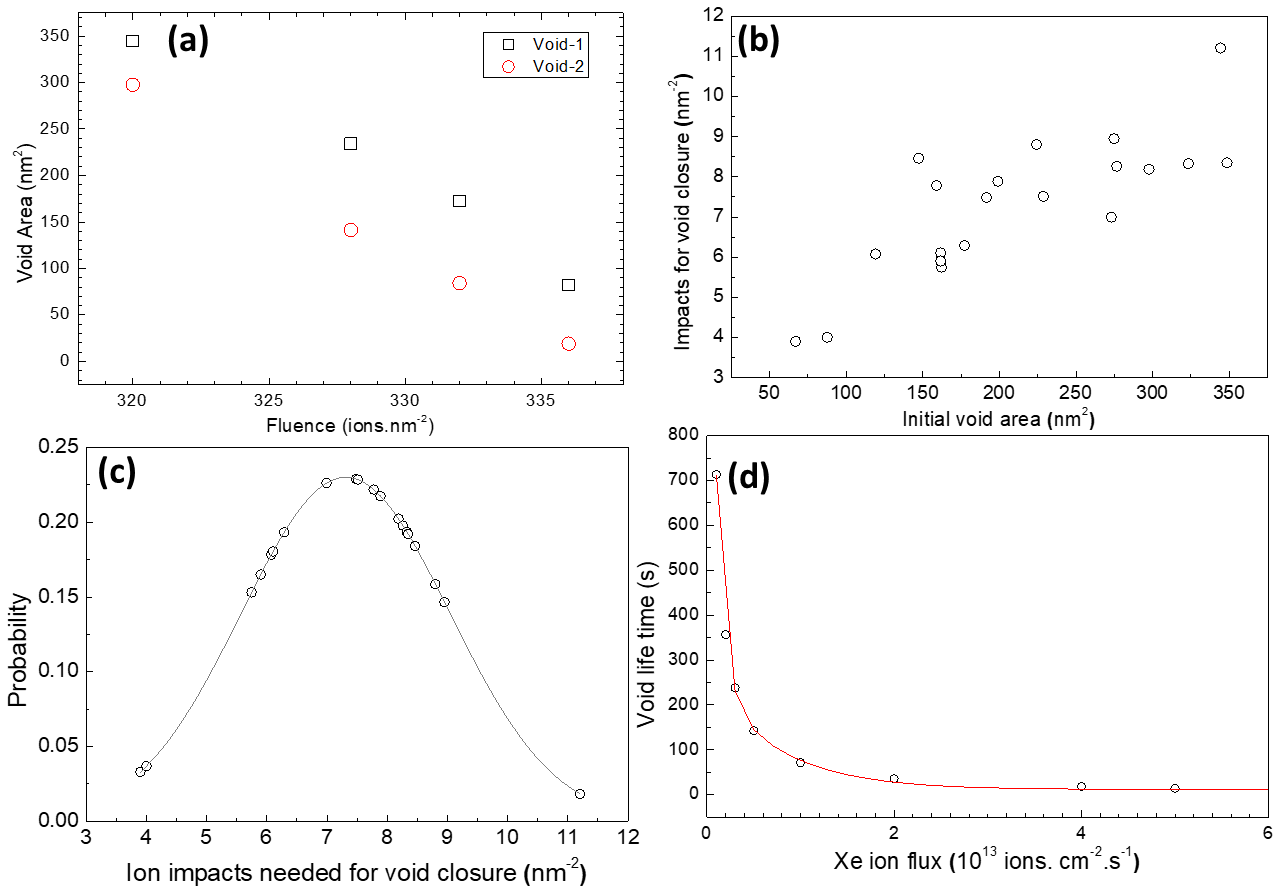


***Figure S3:*** *Behaviour of voids and precipitates under 40 keV Xe ion implantation of a-SiO_2_.* ***(a)****, Decrease of the void area as a function of Xe ion fluence (only two voids shown as an example).* ***(b)****, Number of ion impacts needed per square nanometre of the void for void closure. It was obtained from (a) by integrating the total number of ion impacts received in going from an initial void size to zero void size.* ***(c),*** *Normal distribution fitting of the data in (b) giving a mean of 7±2 ion impacts per square nanometre of the void area.* ***(d),*** *Average time it takes for a void to close depending on the Xe implantation flux.*

------------------------------------------------------------------------------------------------------------------------------------------------------

***Figure S4:*** *MD simulation of effects of 4 keV Xe cascades on solubility sites in a-SiO_2_. The solubility sites for He, Ne and Xe are shown by squares, circles and triangles respectively (obtained by using Delaunay tessellation).*

**Additional details of MD results:** The number of He solubility sites in pristine a-SiO_2_ are predicted in agreement with low-pressure solubility data, but are about an order of magnitude smaller than recent high-pressure solubility data [2]. Ne solubility sites are underestimated by an order of magnitude even when comparing to low-pressure solubility data. Anyway, implantation increased He and Ne solubility sites by a factor of 2 and 5 respectively. No Xe solubility sites were detected in pristine a-SiO_2_ due to low statistics, but implantation caused an overall increase. Relative to the number of Xe solubility sites available after 20 to 50 cascades, there is an increase by a factor of 4 by the end of 300 cascades. The quantitative values are however questionable and improvements in modelling are required from this perspective.

------------------------------------------------------------------------------------------------------------------------------------------------------

***Figure S5:*** *Variation of equilibrium pressure and precipitate density (at 295 K) as a function of precipitate radius. The lines are double exponential function fits to guide the eyes.*

------------------------------------------------------------------------------------------------------------------------------------------------------


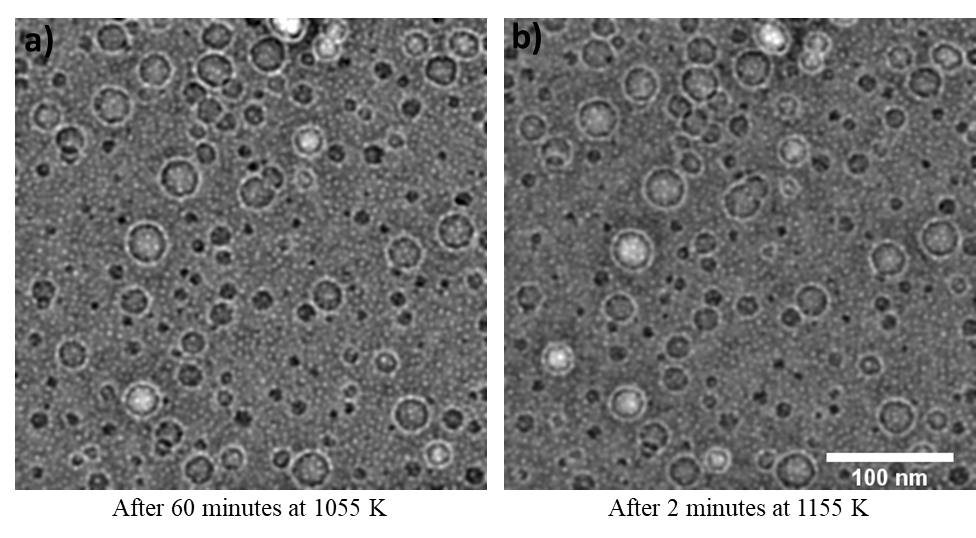


***Figure S6****: Effect of annealing on Xe precipitates formed in a-SiO_2_ after implantation with 40 keV Xe ions.* ***(a, b)****, Xe precipitates (dark and light features) and voids (bright spots) after 60 minutes of annealing at 1055 K and 2 minutes of annealing at 1155 K respectively. Images were taken in a region different from the one shown in the main article. This was necessary as specimen bending in the first region lead to degraded image quality. Note that smaller the precipitates, the darker they are - implying dense precipitates are more stable at higher-temperature (Image defocus = 2 µm over-focus).*

------------------------------------------------------------------------------------------------------------------------------------------------------


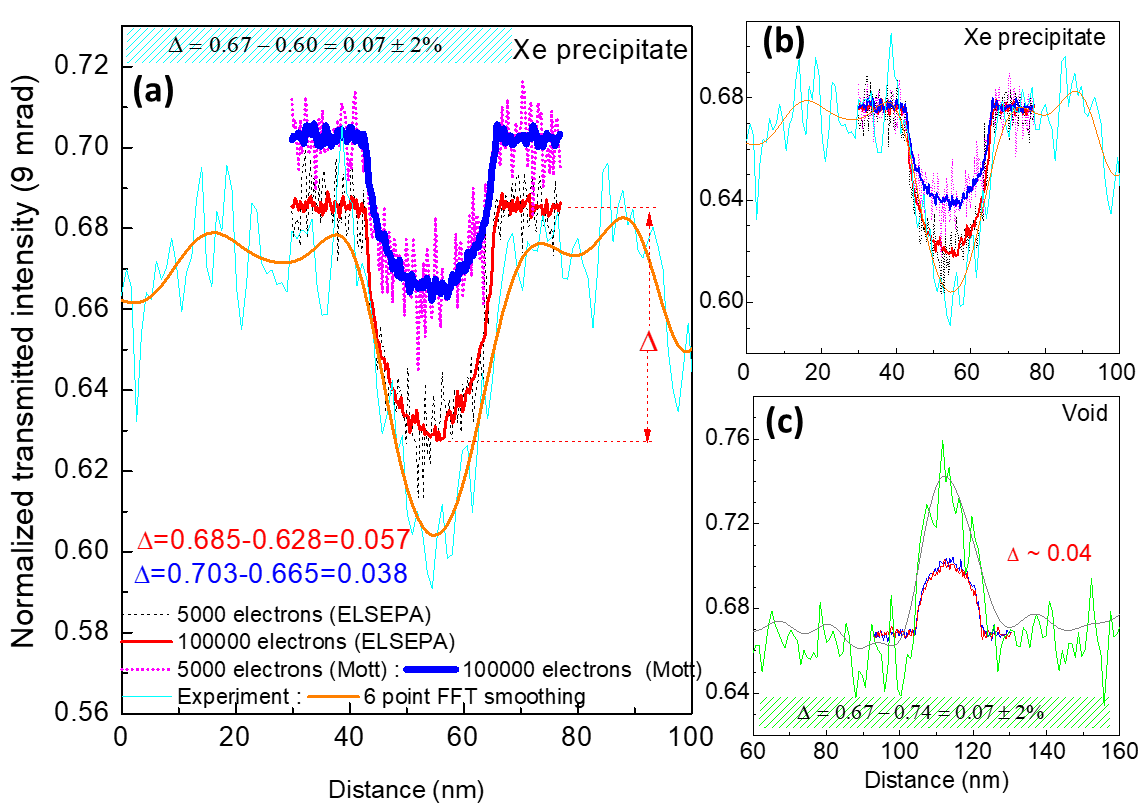


**Figure S7:** *Monte-Carlo simulations of electron transmission through a-SiO_2_ containing xenon precipitates and voids using ELSEPA and Mott electron scattering cross-sections. (****a)****, Experimental transmitted electron intensity profile (in cyan with FFT smoothed signal in orange) (see main article for details). The intensity profiles in black (dotted) and red (solid) are simulated line scans for 5000 electrons per spot and 100000 electrons per spot respectively using ELSEPA cross sections. The intensity profiles in pink (dash) and blue (thick solid) are simulated line scans for 5000 electrons per spot and 100000 electrons per spot respectively using Mott scattering cross sections. The intensity difference between background and Xe precipitate/void is shown by ∆.* ***(b)****, Simulated intensity profiles shifted vertically for easier visual comparison.* ***(c)****, Experimental (in green) and simulated (blue using Mott, and red using ELSEPA cross-sections, vertically shifted profiles) profiles across a void of 20 nm diameter (see main article for more details).*

**Additional Details:** The simulated profiles were obtained by simulating line scans using Mott scattering cross section with either 5000 electrons per spot (pink dashes) or 100000 electrons per spot (thick solid blue), and with ELSEPA scattering cross sections again using either 5000 electrons per spot (black dots) or 100000 electrons per spot (solid red). The line scans simulated using 5000 electrons per spot correspond to image simulation conditions. Line scans using 100000 electrons per spot were simulated for better signal to noise ratio. These line scans are shown overlapped on top of the relatively noisy scans obtained using 5000 electrons per spot. The experimental transmitted intensity through a-SiO_2_ is close to 0.67. Simulations give transmitted intensities of about 0.7 and 0.68 using Mott and ELSEPA scattering cross sections respectively. This shows that Mott scattering cross-sections relatively overestimate the electron transmission through a-SiO_2_. The difference between the intensity transmitted through a-SiO_2_ (background) and centre of the Xe-precipitates as shown by red dotted lines in (a) is represented by symbol ∆ with its values also shown on the figures. Experimental intensity profile shows an intensity drop of 7 ± 2 % due to the presence of 23 nm diameter spherical Xe precipitate. Simulations using ELSEPA and Mott scattering cross sections, show an intensity drop of about 5.7 % and 3.8 % respectively. Thus, ELSEPA cross-sections perform better than Mott scattering cross sections, matching very closely with the experimental results.

------------------------------------------------------------------------------------------------------------------------------------------------------

**Section 2: Supplementary Video information**

------------------------------------------------------------------------------------------------------------------------------------------------------

**Supplementary video SV1:**

Specimen = amorphous SiO_2_

Ion energy = 40 keV Xe, Ion flux ~ 3.9 x10^13^ ions.cm^-2^.s^-1^.

Irradiation temperature = 295 K, Electron beam energy = 300 keV.

Exposure time = 0.25 seconds for each video frame.

Original video size=1600x1600 pixels with 4 frames per second (MPEG-4 compression)

Reduced size=512x512 pixels (bilinear interpolation, Converted to 8 bit).

Processing = auto contrast adjustment using Fiji.

Total frames used for video = 129 frames with each frame corresponding to 1 second of irradiation (258x258 pixels region cropped for the current video).

Frames per second in the video = 20.

Approximate fluence for the first frame = 3.47 x10^16^ ions.cm^-2^.

Approximate fluence for the last frame = 3.98x10^16^ ions.cm^-2^.

Defocus ~ 2µm under-focus.

------------------------------------------------------------------------------------------------------------------------------------------------------

**Supplementary video SV2**:

Specimen = Alkali borosilicate glass called SON68 (see table ST1 for composition).

Ion energy = 45 keV Xe, Irradiation temperature = 295 K, Electron beam energy = 300 keV.

Images were captured after small flux increments and stacked into a video. The fluence for each frame in the video is tabulated below in Table SV2T (flux~2.13x10^13^ ions.cm^-2^.s^-1^).

**Table SV2T**.Fluences (ions.cm^-2^) for various frames in video SV2.

| **Frame number** | **Fluence (ions.cm^-2^)** |
| --- | --- |
| 1 | 8.96E+15 |
| 2 | 1.05E+16 |
| 3 | 1.12E+16 |
| 4 | 1.20E+16 |
| 5 | 1.27E+16 |
| 6 | 1.34E+16 |
| 7 | 1.42E+16 |
| 8 | 1.49E+16 |
| 9 | 1.57E+16 |
| 10 | 1.61E+16 |
| 11 | 1.64E+16 |
| 12 | 1.68E+16 |
| 13 | 1.72E+16 |
| 14 | 1.79E+16 |
| 15 | 1.83E+16 |
| 16 | 1.87E+16 |

------------------------------------------------------------------------------------------------------------------------------------------------------

**Supplementary video SV3**: Monte-Carlo simulation of xenon precipitate to void transformation.

Specimen = amorphous SiO_2_.

Thickness =140 nm, Xenon precipitate radius=6 nm.

Spot size =0.6 nm diameter, 0.3 nm apart, Electron per spot = 5000, Electron energy=300 keV

Collection angle=9 mrad, Elastic scattering cross section=ELSEPA

Density=5, 4.7, 4.4, 4.1, 3.8, 3.5, 3.2, 2.9, 2.6, 2.6, 2, 1.7, 1.4, 1.1, 0.8, 0.5, 0.3, 0 g.cm^-3^ starting from the first frame.

------------------------------------------------------------------------------------------------------------------------------------------------------

**Section 3: Supplementary Tables**

------------------------------------------------------------------------------------------------------------------------------------------------------

**Table ST1**: Composition of SON68 glass (mole percent).

| **SiO_2_** | 45.28 | **Ag_2_O** | 0.03 | **Cs_2_O** | 1.1 | Density=2.75 g cm^-3^ |
| --- | --- | --- | --- | --- | --- | --- |
| **B_2_O_3_** | 13.97 | **CdO** | 0.03 | **TeO_2_** | 0.23 | R=0.72 |
| **Na_2_O** | 10.1 | **Cr_2_O3** | 0.52 | **SnO_2_** | 0.02 | K=3.24 |
| **Al_2_O3** | 4.94 | **ZnO** | 2.5 | **Y_2_O_3_** | 0.2 | Hardness ~6.4 GPa |
| **CaO** | 4.02 | **P_2_O5** | 0.29 | **La_2_O_3_** | 0.92 | Glass transition = 765 |
| **Li_2_O** | 1.97 | **SrO** | 0.34 | **Ce_2_O_3_** | 0.95 | Melting Temperature =1473K |
| **Fe_2_O** | 2.99 | **ZrO_2_** | 2.72 | **Nd_2_O_3_** | 1.64 |  |
| **NiO** | 0.42 | **MoO_3_** | 1.76 | **Pr_2_O_3_** | 0.45 |  |
| **BaO** | 0.61 | **MnO_2_** | 0.38 |  |  |  |

------------------------------------------------------------------------------------------------------------------------------------------------------

**Table-ST2**: Radius, pressure and density of Xe precipitates at 295 K, and after 30 minutes of annealing at 973 K (see main article for details).

| **At 295 K** | | | | **After 30 minutes at 973 K** | | | Remark |
| --- | --- | --- | --- | --- | --- | --- | --- |
| S. No | Radius (nm) | Pressure (GPa) | Density (g.cm^-3^) | Radius (nm) | Pressure (GPa) | Density (g.cm^-3^) |  |
| 1 | 4.15 | 0.14 | 2.80 | 7.93 | 0.08 | 0.96 |  |
| 2 | 7.30 | 0.08 | 2.60 | 8.04 | 0.07 | 0.86 |  |
| 3 | 6.00 | 0.10 | 2.70 | 11.19 | 0.05 | 0.66 |  |
| 4 | 6.88 | 0.09 | 2.70 | 11.71 | 0.05 | 0.66 |  |
| 5 | 9.73 | 0.06 | 2.50 | **14.10** | **0.04** | **0.56** | **void** |
| 6 | 4.15 | 0.14 | 2.80 | 6.40 | 0.09 | 1.00 |  |
| 7 | 5.06 | 0.12 | 2.75 | 10.79 | 0.06 | 0.70 |  |
| 8 | 11.48 | 0.05 | 2.50 | 18.23 | 0.03 | 0.43 |  |
| 9 | 1.99 | 0.30 | 3.10 | **Not clearly resolved** | | |  |
| 10 | 2.81 | 0.21 | 2.08 |  |  |  |  |
| 11 | 2.81 | 0.21 | 2.08 | 3.02 | 0.20 | 1.67 |  |
| 12 | 4.44 | 0.14 | 2.80 | 6.20 | 0.10 | 1.10 |  |
| 13 | 3.29 | 0.18 | 2.85 | 4.15 | 0.14 | 1.40 |  |
| 14 | 8.81 | 0.07 | 2.55 | 14.76 | 0.04 | 0.60 |  |
| 15 | 6.88 | 0.09 | 2.70 | 12.28 | 0.05 | 0.66 |  |
| 16 | 9.50 | 0.06 | 2.50 | 13.88 | 0.04 | 0.60 |  |
| 17 | 8.99 | 0.07 | 2.55 | **12.54** | **0.05** | **0.66** | **void** |
| 18 | 3.58 | 0.17 | 2.85 | 4.86 | 0.12 | 1.25 |  |
| 19 | 4.15 | 0.14 | 2.80 | 5.06 | 0.12 | 1.25 |  |
| 20 | 4.44 | 0.14 | 2.80 | 8.22 | 0.07 | 0.86 |  |
| 21 |  |  |  | **Not clearly resolved** | | |  |
| 22 |  |  |  | 7.45 | 0.08 | 0.95 | **Seen after annealing** |
| 23 |  |  |  | 3.58 | 0.17 | 1.55 |  |
| 24 |  |  |  | 6.88 | 0.09 | 1.00 |  |
| 25 |  |  |  | 7.16 | 0.08 | 0.95 |  |
| 26 |  |  |  | 4.86 | 0.12 | 1.25 |  |
| 27 |  |  |  | 5.79 | 0.10 | 1.10 |  |
| 28 |  |  |  | 2.81 | 0.21 | 1.70 |  |

The entries in bold are voids rather than precipitates. Precipitates numbered from 21 to 28 were seen after annealing. They were either invisible or faintly visible at 295 K.

------------------------------------------------------------------------------------------------------------------------------------------------------

**Table ST3**: Estimation of total implanted Xe into a-SiO_2_.

**Additional Details:**

Implanted fluence =2x10^16^ ions.cm^-2^***.***

Precipitate free volume = V_pf_ = (Volume under implanted Xe profile) **minus** (Volume occupied by precipitates).

Number of Xe atoms in precipitate free volume = (V_pf_) x (Xe solubility limit in irradiated SiO_2_) = V_pf_ x (4x10^21^).

------------------------------------------------------------------------------------------------------------------------------------------------------

**Table ST4**: Calculations based on Isochoric and Isobaric assumption of temperature rise on Xe precipitates. Various evaluated quantities shown in the table are detailed below:

| **Precipitate radius at 295 K (nm)** | **Pressure (Mpa)** | **Density (g.cm^-3^)** | **Temperature (K)** | **Over-pressurization under Isochoric assumption (%)** | **Fractional density drop under Isobaric condition** | **Expected radius based on Isobaric assumption (nm)** | **Observed Radius (nm)** | **Actual density (g.cm^-3^)** | **Observed radius/Expected radius** | **Actual density drop** |
| --- | --- | --- | --- | --- | --- | --- | --- | --- | --- | --- |
| 3.3 | 182 | 2.85 | 295 |  |  |  |  |  |  |  |
|  | 660 | 2.85 | 973 | 262.6 |  |  |  |  |  |  |
|  | 182 | 1.74 | 973 |  | 0.39 | 3.9 | 4.2 | 1.4 | 1.07 | 0.51 |
| 8.8 | 68 | 2.55 | 295 |  |  |  |  |  |  |  |
|  | 481 | 2.55 | 973 |  |  |  |  |  |  |  |
|  | 68 | 0.93 | 973 | 607.4 | 0.64 | 12.3 | 14.8 | 0.6 | 1.20 | 0.76 |
| 6 | 100 | 2.7 | 295 |  |  |  |  |  |  |  |
|  | 562 | 2.7 | 973 |  |  |  |  |  |  |  |
|  | 100 | 1.22 | 973 | 462.0 | 0.55 | 7.8 | 11.2 | 0.7 | 1.43 | 0.76 |

**Additional Details:** Over-pressurization is the difference of pressures at 973 K and 295 K under Isochoric assumption (e.g. for the 3.3 nm precipitate, % Over-pressurization = (660 MPa – 182 MPa)/182 MPa = 262.6 %).

Fractional density drop under isobaric condition is the density decrease in going from 295 K to 973 K assuming Isobaric conditions (e.g., for the 3.3 nm precipitate, fraction density drop under Isobaric condition = (2.85-1.74)/2.85 = 0.39). Expected precipitate radius based on isobaric condition is calculated as follows:

$$\frac{|\Delta\rho|}{\rho}= \frac{\rho_{(295 K)}-\rho_{(973 K)}}{\rho_{(295K)}}=\left( \frac{\frac{M_{1}}{\frac{4}{3}\pi R_{1}^{3}}-\frac{M_{2}}{\frac{4}{3}\pi R_{2}^{3}}}{\frac{M_{1}}{\frac{4}{3}\pi R_{1}^{3}}} \right)$$

Since Mass is conserved ($M_{1}=M_{2}; 1 and 2 are indices for 295 K and 973 K respectively$), after rearrangement:

$$R_{2}^{3}=\left( \frac{R_{1}^{3}}{1-\frac{|\Delta\rho|}{\rho}} \right)$$

Since R_1_ is known from the experiment and $\frac{|\Delta\rho|}{\rho}$ was calculated under Isobaric condition, R_2_ (referred as expected radius in the table) can be evaluated using above equation.

Actual density is the density at 973 K calculated based on the experimental radius observed at 973 K.

------------------------------------------------------------------------------------------------------------------------------------------------------

**Section 4: Supplementary notes and literature**

------------------------------------------------------------------------------------------------------------------------------------------------------

**4.1. Electron scattering**

Taking into account the screening of nucleus by electrons and relativistic effects, Rutherford cross-section for elastic scattering is given as [3]:

$$\sigma_{R}\left( \theta\right)=\frac{Z^{2}\lambda_{R}^{4}}{{64.\pi}^{4}a_{0}^{2}}.\frac{d\Omega}{\left( \sin^{2}\left( \frac{\theta}{2} \right)+\frac{\theta_{0}^{2}}{2} \right)^{2}} (1)$$

Where $\sigma_{R}\left( \theta\right)$ is angle dependent Rutherford elastic scattering cross-section, Z is atomic number of the scattering atom, λ_R_ is relativistic electron wavelength, a_0_ is Bohr radius of hydrogen atom (~0.1 A), $d\Omega$ is the solid angle, θ is scattering angle and θ_0_ is screening parameter. The total scattering cross-section ($\sigma_{total}$) can be obtained by integrating equation (1) from θ =0 to θ =π. The dependence on Z^2^ makes high-Z elements very efficient scattering centres.

The total number of scattering events ‘N_s_’ that a projectile can suffer depends on the probability of scattering (i.e. cross section σ), and total number of scattering centres that the projectile encounters. The latter is proportional to atomic number density (N) and the distance ‘L’ over which such scattering centres are available (i.e. path length). This is given below:

$$N_{s}=\sigma_{total}.N.L$$

The quantity $\sigma_{total}.N$ has the units of inverse of length (m^-1^) and gives total number of collisions suffered per unit length. Its inverse, which represents average distance between two collisions, is called as elastic mean free path (λ).

A comparison of elastic scattering cross-sections of some of the elements relevant to the present scenario is given in Fig. S4.1 (Based on the work of Salvat et al [4] and data distributed by NIST). Figure S4.1 (a) shows differential elastic cross-sections and Fig. S4.1 (b) shows total elastic cross-sections (black squares) and cross-sections integrated up to 8.5 mrad (red circles), used as TEM image collection angle. The inset in Fig. S4.1 (b) shows the total number of collisions suffered by 300 keV electrons while passing through various materials. The noble gas densities considered for this case correspond to 300 MPa of pressure, typical of a 4 nm diameter precipitate. It shows the Xe among all the noble gases would appear darkest, while as He would appear brightest on SiO_2_ background. As ‘N’ decreases with a decrease in the density, the contrast will follow the same trend as observed during the annealing experiment described in the main article.


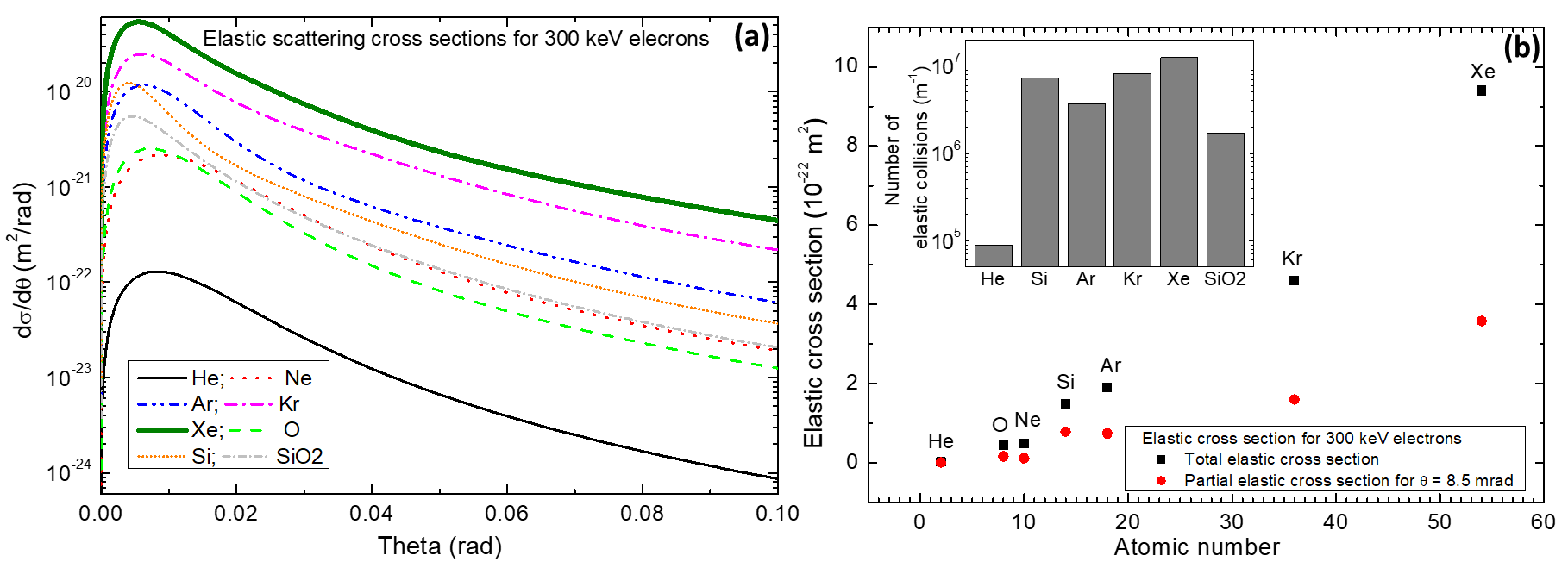


***Figure S4.1:*** *Elastic scattering cross-section of various elements for 300 keV electrons.* ***(a),*** *Differential elastic scattering cross-section of various noble gases, oxygen and silicon for 300 keV electrons. Cross-section of SiO_2_ is atomic fraction weighted cross-section of silicon and oxygen.* ***(b)****, Total elastic cross-section (black squares) and partial elastic cross-section (~8.5 mrad, red circles) as a function of atomic number. The inset shows the total number of elastic collisions suffered by 300 keV electrons from various noble gas precipitates SiO_2_ constituents, and SiO_2_.*

**4.2. MD simulation details**

***Glass preparation:*** The BKS potential [1] was used to simulate the amorphous silica by classical molecular dynamics (DLPOLY code). The interatomic potential applied for a pair of atoms *i-j* separated by a distance *r_ij_* has the following analytical form:

$$\Phi_{ij}\left(r_{ij}\right)=\frac{q_{i}q_{j}}{r_{ij}}+A_{ij}\exp\left(-\frac{b_{ij}}{r_{ij}}\right)-\frac{c}{r_{ij}^{6}}$$

A box containing 99999 atoms (33333 Si and 66666 O) was simulated by applying the following scheme. First, an initial structure was prepared by randomly placing the atoms inside a 114.7 * 114.7 * 114.7 Å^3^ cubic box (2.205g/cm^3^) with the only constraint that two atoms must be separated by at least 1.7Å in order to avoid too intense initial forces. A liquid was equilibrated at 4000 K for 100 ps in the NVT ensemble and then quenched until 300 K at 5x10^12^ K/s. The quenching was performed in steps of 100 K and 20 ps duration. The configuration at 300K was relaxed for 20 ps in the NPT ensemble at ambient pressure to determine the new equilibrium volume. A final equilibration was performed in the NVE ensemble for 5 ps using the equilibrium volume determined previously to relax the atomic configuration. The density of the relaxed silica is equal to 2.22 g.cm^3^.

***Displacements cascade simulation.*** The simulated silica was subjected to a series of 300 displacement cascades to study the ballistic effects. To correctly represent the short-range interactions that can occur during a displacement cascade, ZBL potentials (Ziegler, Biersack, Littmarck) were used for interatomic distances shorter than 0.9Å [3]. The connection between ZBL and BKS potentials was made using polynomial expressions fitted to guarantee continuity of energy and its first two derivatives. To initiate the displacement cascades, 8 Si atoms were modified by giving them the mass of a xenon atom without modifying the interatomic potential. Each projectile is accelerated one after the other with energy equal to 4 keV. During a displacement cascade, the time step was adjusted to have atomic displacements of 0.05Å during one step. Therefore, the time step was small at the beginning and progressively increased until 1fs at the end of the cascade simulation. Three hundred displacement cascades were accumulated keeping the volume fixed. In order to follow the volume change during dose accumulation, an intermediate configuration was relaxed at constant pressure to estimate its density after every 20 cascades.

**4.3. Gas release and the structure of voids.**

Apart from assuming that precipitates turn into voids by losing their confined Xe to the vacuum under de-pressurization, there are two other mechanisms that can lead to de-densification of Xe precipitates and the appearance of void-like features. One possibility is the redistribution of Xe into the surrounding bulk and the other is the formation of multiple closed nano-cracks/nano-channels able to accommodate the Xe from the precipitates. For the first case, consider a Xe precipitate of 5 nm radius at 295 K. Such a precipitate has a Xe density of 1.25x10^22^ atoms.cm^-3^ and will contain a total of about 6790 Xe atoms. Further, assume that the surrounding bulk is completely devoid of any Xe and thus can be loaded with Xe to its maximum limit (which is over exaggeration because the bulk also contains Xe atoms; although at a lower density). Based on the Xe solubility limit of about 4x10^21^ atoms.cm^-3^ in implantation-damaged a-SiO_2_, each Xe on average requires an effective volume of about 2.5x10^-22^ cm^3^. Therefore, a homogeneous redistribution of 6790 atoms into the bulk surrounding the precipitate would require about 1.7x10^-18^ cm^3^ (=6790 x 2.5x10^-22^). The outer radius of the spherical shell centred around the precipitate can be given as:

$$R_{outer}^{3}=R_{inner}^{3}+\frac{3.\pi.V_{Xe}.N_{Xe}}{4}$$

Where R_inner_ is the radius of the precipitate, V_xe_ is effective volume occupied by each Xe atom (~2.5x10^-22^ cm^3^), and N_xe_ is the number of Xe atoms (=6790). This requires a spherical shell of 18 nm outer radius to accommodate all the Xe atoms. From the videos of precipitate to void transformation, we observed that it usually takes 0.25 to 0.5 seconds for complete gas release from the precipitates (going from dark to bright contrast). Redistribution of Xe into a spherical shell of 18 nm during such a short time period is therefore inconceivable under a pure diffusive process due to very low Xe diffusion coefficient at 295 K (At 1300 K, Xe diffusion coefficient in SiO_2_ is about 1x10^-13^ cm^2^.s^-1^ [5]. Room temperature extrapolation gives a diffusion coefficient of about 10^-53^ cm^2^.s^-1^. Even at 900 K, Xe atoms will hardly move by 5 Å during the course of entire ion irradiation).

The other possibility one could assume is the formation of multiple sub-nanometre closed cracks such that Xe diffuses into such cracks. If the cracks were approximated as cylinders of about 1 nm radius, and the atomic density in such cracks was assumed to be at most of the same level as that in the precipitate itself (the atomic density would in fact be less), then a cumulative crack length of about 170 nm would be required to accommodate all the Xe atoms. Since no cracks were observed radiating from the precipitates in the TEM images, the only possibility is if such cracks were oriented perpendicular to the specimen surface. In fact, it is possible that such cracks or nano-channels may help in temporarily connecting the precipitates to the vacuum and lead to depressurization and Xe loss. Nonetheless, from the live videos (captured at four frames per second) precipitate to void transformation was observed to take place generally in less than 0.25 to 0.5 s. A precipitate of 20 nm diameter will contain about 4.7×10^4^ Xe atoms under equilibrium conditions. Transformation into a void of such a precipitate in 0.25 to 0.5 s would require gas release rates of about 9.4×10^4^ to 1.9×10^5^ atoms.s^–1^. This corresponds to a gas release rate of at least 3.4×10^–15^ to 6.8×10^–15^cm^3^.STP.s^–1^ (about 2×10^–17^ to 4×10^–17^ grams per second; STP stands for standard temperature and pressure). Due to a lack of the data on Xe release rates through glass nanochannels, we cannot deduce the nanochannel sizes required for such gas release rates under depressurizations of about 100 MPa; which most of the precipitates should experience. However, future studies may shed some light on this aspect.

Another interesting question is whether the voids themselves are open structures such as: surface craters; blisters left after gas release; or closed structures underneath the surface which are temporarily connected to the outside vacuum during the gas loss. Although the current work offers no direct answer, the complete filling of all the voids during the early stages of annealing indicates that the most likely explanation is that the voids are closed structures which temporarily open to the vacuum during ion implantation.

**4.4. Notes and some literature on the solubility**

Maximum Xe solubility limit of 6 ± 1.7 atomic % (~4x10^21^ ions.cm^-3^) and 2 ± 0.6 atomic % (~1.7x10^21^ atoms.cm^-3^) were obtained for implanted a-SiO_2_ and SON68 glasses respectively (shown in Fig. S4.4 (a, b) as SiO_2_-irradiated and SON68-irradiated). MD simulations of 4 keV Xe cascades in a-SiO_2_ showed implantation effects can increase the Xe solubility by about 40% in a-SiO_2_. Assuming a similar effect taking place in SON68 glass, xenon solubility in pristine a-SiO_2_ and pristine SON68 glass can be expected to be 40 % smaller. By considering 40 % lower solubility for pristine SiO_2_ and pristine SON68, corrected values are shown as “SiO2-MD corrected “and “SON68-MD corrected” in Fig. S4.4 (a, b). A lower Xe solubility in SON68 glass is expected due to the presence of about 16% alkali and alkaline network modifiers and only about 60% network formers. It is generally agreed based on experimental [6–11] as well as on numerical studies [12] that network modifying cations occupy some of the available solubility sites. This reduces the number of sites accessible to gases, thereby, decreasing the solubility limit (See for instance Table 5 and Fig. 1 in [6] to get an idea of the variation of gas solubility with a change in the modifier content in binary and ternary silicate glasses). Therefore, ion irradiation results are in good qualitative agreement with these findings. A precise quantitative comparison with the solubility data available in the literature is difficult partly due to lack of high-pressure solubility data for Xe, and partly due to large variations in the available solubility limits. What follows next is a discussion and comparison of some of the solubility data available in literature and where ion irradiation results stand with respect this data.

To the best of our knowledge, there is no experimental data on high-pressure (~few GPa) Xe solubility in a-SiO_2_. Some high-pressure experiments (up to 12 GPa) of Ar and Xe solubility in Tholeiitic and Haplogranitic glass compositions have shown that solubility increases fairly linearly with pressure before saturating at 4 to 6 GPa [7]. Also, numerical simulations of He, Ne, Ar and Xe solubilities in Rhyolite, MORB (Mid Oceanic Ridge Basalt) and Olivine have shown that noble gas solubilities increase linearly with pressure and saturate after 2 to 5 GPa [13] (lower pressure for larger atom). Numerical simulations of Ar solubility in a-SiO_2_ show a linear increase up to 10 GPa, where a saturation solubility of about 11 weight percent (~3.8x10^21^ atoms.cm^-3^) is attained [13]. Recent high-pressure He solubility experiments on a-SiO_2_ also show a linear increase with pressure with a slow tendency towards saturation in 5 to 10 GPa range after attaining a solubility of about 2x10^22^ to 5 x10^22^ atoms .cm^-3^. Solubility limits derived from these high-pressure experiments and numerical simulations clearly show that solubility limits obtained from low-pressure data can be underestimated by at least a factor of two to more than an order of magnitude (for instance; 2x10^21^ He.cm^-3^ from low-pressure [10] Vs 2x10^22^ He.cm^-3^ from high-pressure [2] in a-SiO_2_, 1x10^20^ Ar.cm^-3^ Vs 2 x10^21^ to 4x10^21^ Ar.cm^-3^ from high-pressure in a-SiO_2_ [13,14] or underestimates by up to a factor of 4 in some other glasses). Based on these limited high-pressure experiments and numerical simulations, we assume that solubility in general saturates after about 5 GPa. Therefore, the solubility data available in the literature mostly in cm^3^ STP.g^-1^.bar^-1^ is expressed at 5 GPa in atoms.cm^-3^ and is shown in Fig. S4.4 (a, b) (See legend for references). The solubility of Ar in a-SiO_2_ obtained this way (~3.3x10^21^ atoms. cm^-3^) is in very good agreement with the values expected from numerical simulations (~3.8x10^21^ atoms.cm^-3^). Similarly, solubility of He in a-SiO_2_ obtained this way (~1.6x10^22^) is in reasonable agreement with the lower limit of recent high-pressure He solubility values ranging from 2x10^22^ to 5x10^22^ atoms.cm^-3^ in 5 to 10 GPa range (shown as SiO_2_-High-pressure in Fig. S4.4 (a)) [2]. From Ar and Xe solubility in Rhyolite (which is about 80% SiO_2_), and many sodium silicate glass compositions listed in [6] (one of which written as Na_2_O-SiO_2_ is shown in Fig. S4.4 (a, b)), we calculated that Xe solubility is about 3 to 4 times lower than Ar solubility. We therefore assume a similar factor of solubility drop should occur in a-SiO_2_ in going from Ar to Xe. Ar solubility in SiO_2_ at 5 GPa divided by 3 is therefore regarded as the expected Xe solubility in a-SiO_2_ (shown as SiO2-expected in Fig. S4.4 (b) ~ 1x10^21^ atoms.cm^-3^). In comparison, the solubility derived from ion implantation and corrected for radiation damage using MD studies is about 2.4x10^21^ (± 30%). In light of the large uncertainties associated with the solubility data available in the literature, it is not an exaggeration to say that this value reasonably matches with the predicted solubility. In addition, we showed that Xe present in the precipitates and in the solubility sites correctly account for the total implanted Xe, thereby proving further confidence on this estimate.

Nevertheless, some other perspectives on this subject are worth a consideration. Low-pressure solubility data of olivine when expressed at 5 GPa seem to indicate a Xe solubility of about 8x10^18^ atoms.cm^-3^ (shown as Olivine (5 GPa) in Fig. S4.4 (a, b)). However, recent DFT calculations [15] where the authors also reanalysed some previous high-pressure Xe infusion experiments (2 to 5 GPa) [16] showed that Xe solubility in olivine can be as high as 3x10^20^ atoms.cm^-3^. This is a factor of 40 increase with rest to previous estimates and coherent with earlier examples of a-SiO_2_. The reason behind this large Xe solubility in Olivine has been hypothesized to be Si substitution by Xe leading to the formation of xenon oxide (XeO_2_) which is predicted to be stable at ambient temperature and pressure [15–17]. Also, formation of XeO_2_ in planar square geometry in silica glass fibres has been predicted by numerical simulations [17]. As to how such Xe-silica linear structures can form under geological conditions remains an open question [17] (There is however a possibility of this happening under irradiation environment as we discuss later). Thus, a combination of chemical and physical solubility can result in higher solubility limits.

From a number of ion irradiation studies, it is known that radiation damage in simple and complex oxide glasses saturates after an energy deposition of about 2x10^20^ keV.cm^-3^ [18]. Using a 40 keV Xe ion, it will be reached after implantation with about 2x10^13^ ions.cm^-2^. Assuming each ion produces about 10^8^ Si vacancies per cm per ion (based on SRIM simulations [1]), the total number of Si vacancies can be expected to be about 2x10^21^ cm^-3^. A part of such vacancies may then be occupied by implanted Xe ions (which may require local network distortion to accommodate the Xe atom) resulting in a combination of physical and chemical solubility. However, the knocked-out Si atoms on the other hand will occupy interstitial sites, thereby decreasing the number of physical solubility sites available to Xe atoms. Since Si atomic radius (0.41 A) is less than Vander walls radius of any of the noble gases, and He solubility sites are in general an order of magnitude higher than Xe solubility sites, Si can be expected to predominantly reside in the lower side of the solubility site distribution. Presence of Si interstitials therefore may not have a significant effect on physical Xe solubility limit, whereas Si vacancies may provide a way for chemical solubility of Xe. The net effect of this Xe/Si substitution and formation of Si interstitials may therefore end up slightly increasing the total Xe solubility. Current numerical simulations however put planar geometry constraint for Xe incorporation into the glass network. This constraint would thus reduce the feasible Si vacancy sites. Interestingly, using Raman spectroscopy, we have shown in our previous ion irradiation studies of a-SiO_2_, that radiation damage can result in the formation of linear silica chains or possibly highly strained two-member silica rings [19]. Therefore, although the question regarding the incorporation of Xe gas into fibrous silica remains an open, Radiogenic Xe on the other hand can be accommodated into the silica chains created during self-ion irradiation. Thus, a factor of 2 overestimation in Xe solubility from implantation studies may be reminiscent of something more fundamental taking place that is worth further investigation.


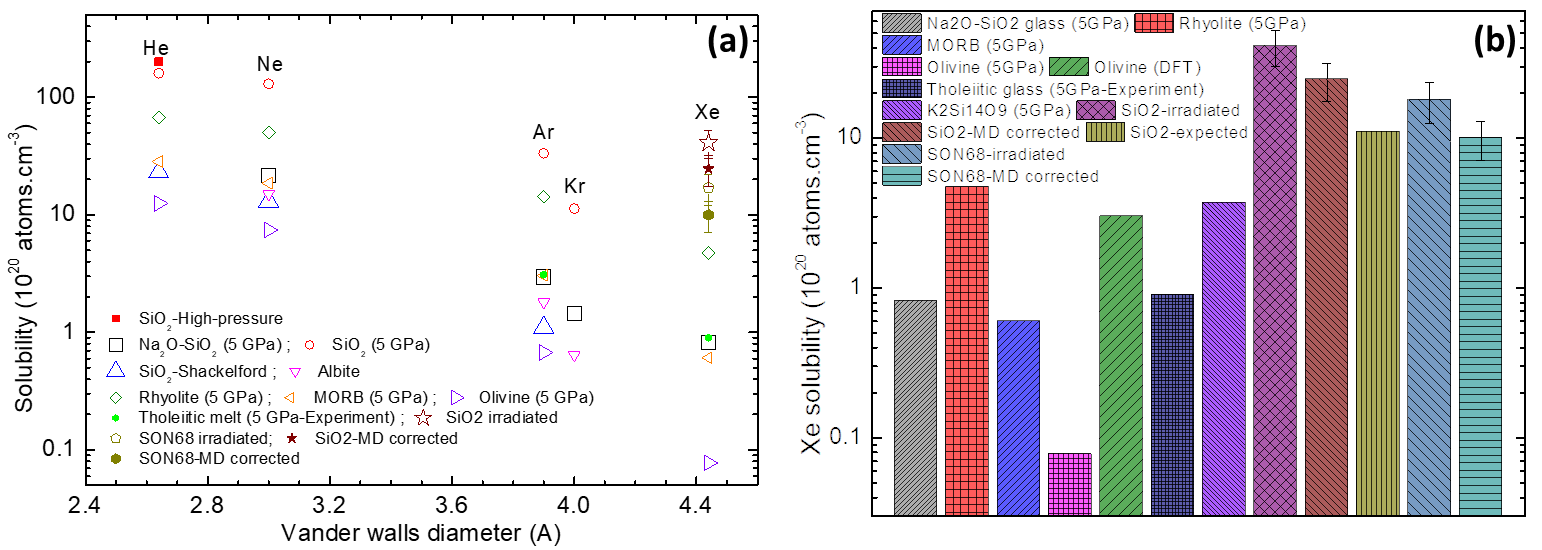


***Figure S4.4.*** *Solubility of noble gases in various materials.* ***(a)****, Solubility in atoms.cm^-3^ as a function of Vander walls diameter. The original solubility data expressed mostly in cm^3^ STP.g^-1^.bar^-1^ was multiplied by 5 GPa pressure and then converted to atoms.cm^-3^ (1 cm^3^ STP=2.7x10^19^ atoms). The original data is from following references: Na_2_O-SiO_2_* [6] *(Table 5, NS1, G205 data set), SiO_2_* [20] *(Fig. 5 and 6)* [8] *(Table 3), SiO2-Shackelford* [10] *(Table 1),SiO_2_ high-pressure*[2] *(Figure 5), Albite* [8] *(Table 3), Rhyolite, MORB, Olivine* [13] *(data at 2000 K from Fig. 1), Tholeiitic melt* [7] *(Fig. 2 and 3. These are high pressure experiments), Rest of the data points (this work, see text for details).* ***(b)****, Solubility of Xe in various materials. Most of the data in this figure is from Fig. (a). SiO_2_-expected was obtained by dividing Ar solubility by 3 (see text for details). Some additional data in this figure is from following references: Olivine (DFT)* [15]*, K_2_Si_14_O_9_* [9]*.*

**4.5. Additional pre-requisites for Monte-Carlo simulations**

*The range of angles from which scattered electrons were collected.* This collection angle is controlled by the size of the objective aperture in a TEM (8.5 mrad in the current work). The CASINO code allows increments of 1 mrad only. Therefore, a comparison of the experimental TEM images of a-SiO_2_ captured using 8.5 mrad collection angle with images simulated using 9 mrad is made. Auxiliary simulations (not shown) at 8 mrad did not reveal any significant differences in comparison to the 9 mrad case. Therefore, the effect of the 0.5 mrad increase in collection angle in the simulations is considered to be negligible.

*Elastic scattering cross-sections.* The CASINO software allows choosing from different elastic scattering cross-sections. A comparison of the results based on Mott and ELSEPA scattering cross-sections has been performed and is presented in the SI. Based on the agreement between the experimental and simulated transmitted intensities (see Fig. S7 in the SI), ELSEPA cross-sections were regarded to be better than the Mott scattering cross-sections and were thus used for the image simulations presented here.

**4.6. Xe density and Xe equations of state**

The pressures shown in table ST2 range from 52 MPa for a 22 nm diameter precipitate to 302 MPa for a 4 nm diameter precipitate at 295 K and from 33 MPa for a 35 nm diameter precipitate to 214 MPa for a 5.6 nm diameter precipitate at 973 K. Figure S4.6(a) shows the Xe melting curve [21,22]. The solid curve is a theoretical prediction based on the work of Belonoshko [23] and the dashed line shows the results from Monte Carlo simulations [24]. For solid Xe to exist at 295 K, pressures greater than 423 MPa are needed (Fig. S4.5(a)). This requires precipitate diameters less than 2.8 nm. For precipitate diameters greater than 3 nm, the melting temperature is less than the Xe critical temperature of 289.7 K. Since the Xe critical pressure is 5.8 MPa and all the precipitates listed in Table ST2 in the SI at 295 K (as well as at 973 K) have pressures greater than this.

Although the pressure-density (*P*-*ρ*) data at high-temperatures are scarce in the literature, a few high-pressure studies around room temperature are available [25–29]. Some of this experimental data covering the low-pressure range up to about 100 MPa [25,28] and the high-pressure range above 1 GPa [26–29] are plotted in Fig. 8 (b). The Carnahan-Starling hard-sphere EOS (CS-EOS) [30] — which is particularly suitable for high-temperature or high-pressure cases — using hard-sphere diameters calculated either from the Lenard-Jones potential (LJ) or the modified Buckingham potential are also plotted. In addition, Ronchi’s high-temperature high-pressure extrapolation is also shown. For pressures greater than 20 MPa, Ronchi's extrapolation reproduces the experimentally-measured densities to within 13% (the agreement is much better for lower pressures – see red curve in the inset in Fig. 8(b)). For pressures less than 300 MPa, CS-EOS underestimates the density by about 30 to 40%. However, for pressures greater than 700 MPa various EOSs are very close to each other with the CS-EOS (using the modified Buckingham potential) reproducing the experimental results to within 3%. Figure 8(c) shows density (*ρ*), the first derivative of the density vs. pressure (d*ρ*/d*p*) and the density normalized d*ρ*/d*p* in percent (1/*ρ*.d*ρ*/d*p*) as functions of pressure. This figure clearly shows that due to the relatively-low slope of the density vs pressure curve in the pressure range of greater than a few tens of MPa, an error in pressure calculation even by a factor of two (either due to uncertainties in the surface tension values or due to the use of the capillary equation itself) can lead to only a 20% miscalculation in the density. For pressures greater than a few GPa, the uncertainty is just a few percent. For typical precipitate pressures in this study, a 20% uncertainty in the pressure values will cause an uncertainty of about 3% in the density values. Therefore, 20 to 30% uncertainty in the surface tension values is not significantly detrimental to the density calculation itself. Owing to such uncertainties, it can therefore be assumed that the densities at room temperature have an error of 5 to 10%.

For high temperature (973 K) and pressures (33 to 214 MPa) typical of the annealing experiment performed in this study, we could not find any experimental *P*-*ρ* data in the literature. The densities used in the current work are therefore calculated entirely based on the EOS. The *P*-*ρ* data in the temperature range of 900 to 1000 K are shown in Fig. 8(d) based on the CS-EOS, the Ronchi extrapolation and the Harrison extrapolation [29]. For the Ronchi extrapolation, a comparison of densities at 300, 900 and 1000 K is given. There is no significant density change in going from 900 to 1000 K (a drop of about 0.07 %.K^–1^). Therefore, different EOSs calculated within a few tens of K from the actual annealing temperature of 973 K (e.g. the Harrison extrapolation) are reasonable approximations. A temperature increase from 295 to 973 K decreases the density by about 10% for pressures greater than 1 GPa (about 1.5% per 100 K). The maximum density drop occurs in the initial part of the *P*-*ρ* curve where the slope is relatively large. For pressures up to 1 GPa, various EOSs (except the CS-EOS using the LJ potential) give densities to within 2% of each other. Therefore, any of these EOSs would be equally suitable in this range. Again the CS-EOS with modified Buckingham potential and Ronchi's extrapolation are very close to each other with the CS-EOS giving slightly higher densities for pressures > 2 GPa (by about 5%). This is similar to the behaviour seen at 300 K, where the CS-EOS using the modified Buckingham potential gives slightly higher densities than the Ronchi-extrapolation but closer to the available experimental data shown in Fig 8 (b). Based on this observation, we therefore assume that the CS-EOS again gives a slightly better estimate of the densities at high-temperature. For the precipitate pressures shown in table ST2 in the SI, the densities were calculated using Ronchi’s extrapolation at 1000 K and the CS-EOS using a modified Buckingham potential at 973 K and an average of the two values was taken for the image simulation (the average density values are 6% higher/lower than the CS-EOS/Ronchi extrapolation, respectively). It is important to emphasize that for high pressures (> 1 GPa), choosing between the Harrison extrapolation and other EOSs will lead to appreciably different densities but that this is less of a concern for lower pressures. The uncertainty on the density estimates at best cannot be better than the 10% uncertainty at room temperature. A comparison of the MD results and Ronchi's data for temperatures from 300 to about 2300 K given elsewhere [31] shows that the results agree to within a few % (4 to 8 % in the 0.1 to 1 GPa range). Therefore, we assume that the densities at 973 K should not be in error by more than 10%.


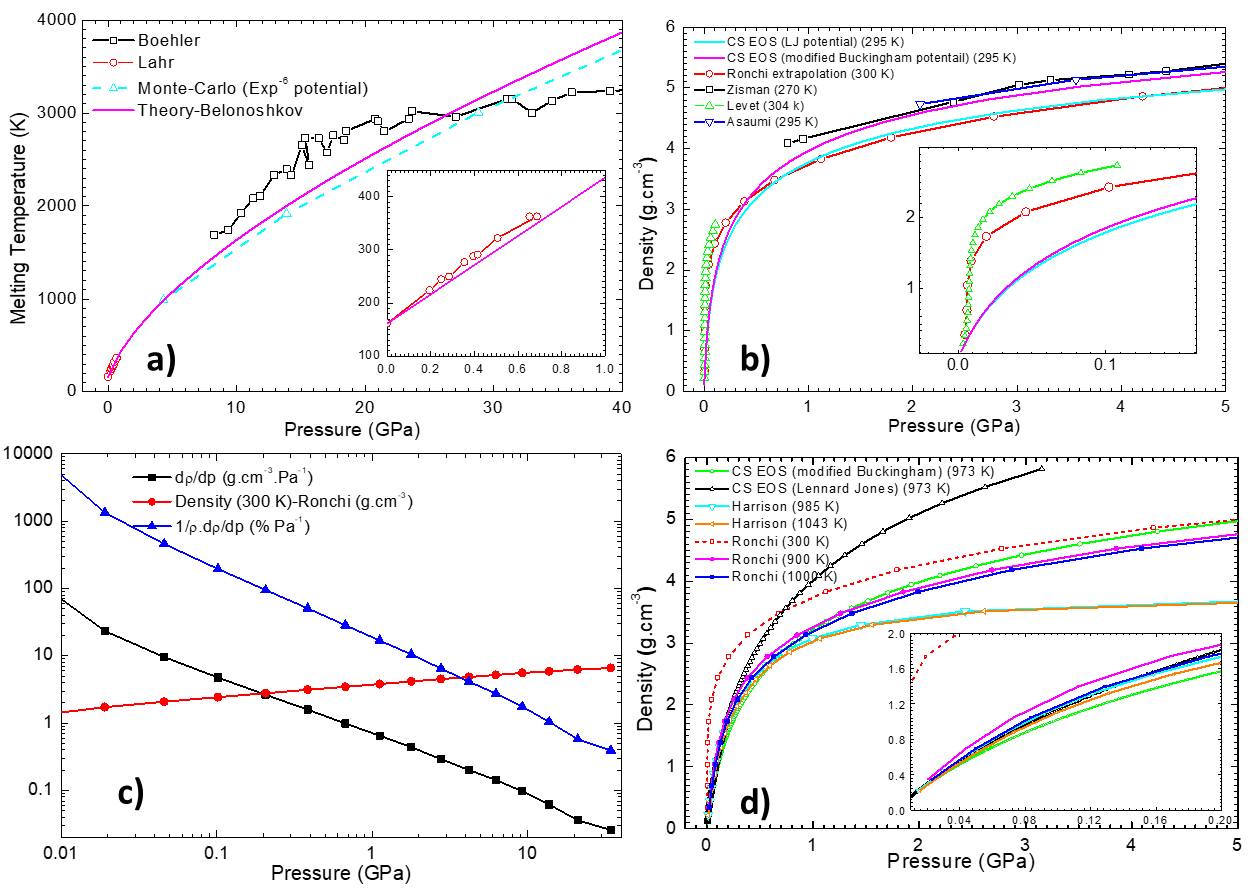


***Figure S4.6****. Pressure dependence of Xe density and melting temperature.* ***(a)*** *variation of Xe melting temperature as a function of the pressure with the inset showing the data in the < 1 GPa range;* ***(b)*** *Xe density as a function of the pressure near room temperature;* ***(c)*** *variation of density (*ρ*), first derivative of the density with rest to pressure (d*ρ*/d*p*) and density normalized d*ρ*/d*p *in percent (1/ρ.dρ/dp) as functions of pressure; and* ***(d)*** *Xe density as a function of pressure at high-temperature.*

The data displayed in the graphs in Fig. 8 is from following references: high-pressure data is from Boehler et al. [21]; low-pressure data is from Lahr et al. [22]; results of Monte-Carlo simulations using exp^-6^ model [24]; theoretical prediction based on the work of Belonoshko et al [23], Zisman et al. [26], Levelt [25] and Asaumi [27]; CS-EOS curves are predictions of Carnahan-Starling EOS [30] employing either Lenard-Jones potential or modified Buckingham potential; Ronchi extrapolation is based on the work of Ronchi [28]; and Harrison’s data is from ref. [29].

------------------------------------------------------------------------------------------------------------------------------------------------------

Ref

[1] J.F. Ziegler, M.D. Ziegler, J.P. Biersack, SRIM – The stopping and range of ions in matter (2010), Nucl. Inst. Methods Phys. Res. B. 268 (2010) 1818–1823. doi:10.1016/j.nimb.2010.02.091.

[2] T. Sato, N. Funamori, T. Yagi, Helium penetrates into silica glass and reduces its compressibility, Nat. Commun. 2 (2011) 345. doi:10.1038/ncomms1343.

[3] D.B. Williams, C.B. Carter, Transmission Electron Microscopy, Springer US, Boston, MA, 2009. doi:10.1007/978-0-387-76501-3_1.

[4] F. Salvat, A. Jablonski, C.J. Powell, Elsepa - Dirac partial-wave calculation of elastic scattering of electrons and positrons by atoms, positive ions and molecules, Comput. Phys. Commun. 165 (2005) 157–190. doi:10.1016/j.cpc.2004.09.006.

[5] K. Roselieb, W. Rammensee, H. Büttner, M. Rosenhauer, Diffusion of noble gases in melts of the system SiO2-NaAlSi2O6, Chem. Geol. 120 (1995) 1–13. doi:10.1016/0009-2541(94)00117-Q.

[6] T. Shibata, E. Takahashi, J. Matsuda, Solubility of neon, argon, krypton, and xenon in binary and ternary silicate systems: A new view on noble gas solubility, Geochim. Cosmochim. Acta. 62 (1998) 1241–1253. doi:Doi 10.1016/S0016-7037(98)00046-5.

[7] B.C. Schmidt, H. Keppler, Experimental evidence for high noble gas solubilities in silicate melts under mantle pressures, Earth Planet. Sci. Lett. 195 (2002) 277–290. doi:10.1016/S0012-821X(01)00584-2.

[8] M.R. Carroll, S.R. Sutton, M.L. Rivers, D.S. Woolum, An experimental study of krypton diffusion and solubility in silicic glasses, Chem. Geol. 109 (1993) 9–28. doi:10.1016/0009-2541(93)90059-R.

[9] Antonio Paonita, Noble gas solubility in silicate melts: a review of experimentation and theory, and implications regarding magma degassing processes, Ann. Geophys. 48 (2005) 647.

[10] J.F. Shackelford, Gas Solubility and Diffusion in Oxide Glasses – Implications for Nuclear Wasteforms, Procedia Mater. Sci. 7 (2014) 278–285. doi:10.1016/j.mspro.2014.10.036.

[11] S.P. FAILE, D.M. ROY, Solubilities of Ar, N2, CO2, and He in Glasses at Pressures to 10 Kbars, J. Am. Ceram. Soc. 49 (1966) 638–643. doi:10.1111/j.1151-2916.1966.tb13190.x.

[12] A. Kerrache, J.M. Delaye, Interstitial sites for He incorporation in nuclear glasses and links to the structure: Results from numerical investigation, Nucl. Instruments Methods Phys. Res. Sect. B Beam Interact. with Mater. Atoms. 326 (2014) 269–272. doi:10.1016/j.nimb.2013.08.075.

[13] B. Guillot, N. Sator, Noble gases in high-pressure silicate liquids: A computer simulation study, Geochim. Cosmochim. Acta. 80 (2012) 51–69. doi:10.1016/j.gca.2011.11.040.

[14] K. Niwa, C. Miyakawa, T. Yagi, J.I. Matsuda, Argon solubility in SiO2 melt under high pressures: A new experimental result using laser-heated diamond anvil cell, Earth Planet. Sci. Lett. 363 (2013) 1–8. doi:10.1016/j.epsl.2012.12.014.

[15] C. Crépisson, M. Blanchard, M. Lazzeri, E. Balan, C. Sanloup, New constraints on Xe incorporation mechanisms in olivine from first-principles calculations, Geochim. Cosmochim. Acta. 222 (2018) 146–155. doi:10.1016/j.gca.2017.10.028.

[16] C. Sanloup, B.C. Schmidt, G. Gudfinnsson, A. Dewaele, M. Mezouar, Xenon and Argon: A contrasting behavior in olivine at depth, Geochim. Cosmochim. Acta. 75 (2011) 6271–6284. doi:10.1016/j.gca.2011.08.023.

[17] J. Kalinowski, M. Räsänen, R.B. Gerber, Chemically-bound xenon in fibrous silica, Phys. Chem. Chem. Phys. 16 (2014) 11658–11661. doi:10.1039/C4CP01355G.

[18] E.P. EerNisse, C.B. Norris, Introduction rates and annealing of defects in ion-implanted SiO2 layers on Si, J. Appl. Phys. 45 (1974) 5196. doi:10.1063/1.1663215.

[19] A.H. Mir, M. Toulemonde, C. Jegou, S. Miro, Y. Serruys, S. Bouffard, S. Peuget, Understanding and simulating the material behavior during multi-particle irradiations, Sci. Rep. 6:30191 (2016).

[20] C.C. Tournour, J.E. Shelby, Inert gas solubility in binary germania–silica glasses, J. Non. Cryst. Solids. 349 (2004) 209–214. doi:10.1016/j.jnoncrysol.2004.08.144.

[21] R. Boehler, M. Ross, P. Söderlind, D.B. Boercker, High-pressure melting curves of argon, krypton, and xenon: Deviation from corresponding states theory, Phys. Rev. Lett. 86 (2001) 5731–5734. doi:10.1103/PhysRevLett.86.5731.

[22] P.H. Lahr, W.G. Eversole, Compression Isotherms of Argon, Krypton, and Xenon Through the Freezing Zone, J. Chem. Eng. Data. 7 (1962) 42.

[23] A.B. Belonoshko, S. Davis, A. Rosengren, R. Ahuja, B. Johansson, S.I. Simak, L. Burakovsky, D.L. Preston, Xenon melting: Density functional theory versus diamond anvil cell experiments, Phys. Rev. B. 74 (2006) 054114. doi:10.1103/PhysRevB.74.054114.

[24] F. Saija, S. Prestipino, High-pressure phase diagram of the exp-6 model: The case of Xe, Phys. Rev. B - Condens. Matter Mater. Phys. 72 (2005) 2–11. doi:10.1103/PhysRevB.72.024113.

[25] J.M.H. Levelt, The reduced equation of state, internal energy and entropy of argon and xenon, Physica. 26 (1960) 361–377.

[26] A.N. Zisman, I. V. Aleksandrov, S.M. Stishov, X-ray study of equations of state of solid xenon and cesium iodide at pressures up to 55 GPa, Phys. Rev. B. (1985). doi:10.1103/PhysRevB.32.484.

[27] K. Asaumi, High-pressure x-ray diffraction study of solid xenon and its equation of state in relation to metallization transition, Phys. Rev. B. 29 (1984) 7026–7029. doi:10.1103/PhysRevB.29.7026.

[28] C. Ronchi, Extrapolated equation of state for rare gases at high temperatures and densities, J. Nucl. Mater. 96 (1981) 314–328. doi:10.1016/0022-3115(81)90575-4.

[29] J.W. Harrison, An extrapolated equation of state for xenon for use in fuel swelling calculations, J. Nucl. Mater. 31 (1969) 99–106.

[30] N.F. Carnahan, K.E. Starling, Equation of State for Nonattracting Rigid Spheres, J. Chem. Phys. 51 (1969) 635–636. doi:10.1063/1.1672048.

[31] J.Y. Oh, Y.H. Koo, J.S. Cheon, B.H. Lee, D.S. Sohn, Molecular dynamics simulation of the pressure-volume-temperature data of xenon for a nuclear fuel, J. Nucl. Mater. 372 (2008) 89–93. doi:10.1016/j.jnucmat.2007.02.009.
